# Oral Fecal Microbiota Transplantation in Dogs with Tylosin-Responsive Enteropathy—A Proof-of-Concept Study

**DOI:** 10.3390/vetsci11090439

**Published:** 2024-09-18

**Authors:** Mohsen Hanifeh, Elisa Scarsella, Connie A. Rojas, Holly H. Ganz, Mirja Huhtinen, Tarmo Laine, Thomas Spillmann

**Affiliations:** 1 Department of Equine and Small Animal Medicine, Faculty of Veterinary Medicine, University of Helsinki, 00790 Helsinki, Finland; thomas.spillmann@helsinki.fi; 2 AnimalBiome, 400 29th Street, Suite 101, Oakland, CA 94609, USA; elisa@animalbiome.com (E.S.); connie@animalbiome.com (C.A.R.); holly@animalbiome.com (H.H.G.); 3 Orion Corporation, R&D, 02200 Espoo, Finland; mirja.huhtinen@orionpharma.com (M.H.); tarmo.laine@orionpharma.com (T.L.)

**Keywords:** chronic enteropathy, tylosin-responsive enteropathy, dogs, fecal microbiota transplant, fecal microbiome, microbiome diversity, placebo

## Abstract

**Simple Summary:**

Antibiotic resistance is a major concern, and dogs with tylosin-responsive enteropathy (TRE) often receive the antibiotic for extended periods. Fecal microbiota transplantation (FMT) could be an alternative treatment. This clinical trial compared the impact of oral FMT/placebo capsules on 14 TRE dogs (7 FMT, 7 placebo) by analyzing their fecal consistencies and gut microbiomes. The microbial engraftment was assessed based on the gut microbiome of the single stool donor. In addition, the gut microbiome of these 14 dogs was compared with the fecal microbial composition of 30 healthy dogs. During the 4-week treatment trial, 5/7 dogs in the FMT group (71.4%) and 3/6 dogs in the placebo group (50%) did not relapse. After the trial treatment and 4-week follow-up, 2 more dogs in the FMT group and 1 dog in the placebo group relapsed, but none in the placebo group. The differences in relapse rates between the FMT and placebo groups were not statistically significant. TRE dogs had lower microbiome diversity when on tylosin, but it increased after treatment with FMT or placebo. On average, 30.4% of the donor bacterial strains were engrafted into FMT recipients. Overall, the FMT efficacy was slightly higher than the placebo, but the difference was not statistically significant, possibly because of the small sample size of the study. Future clinical trials should include larger sample sizes to determine the efficacy of oral FMT in dogs with TRE or chronic enteropathies.

**Abstract:**

A clinical trial was conducted to evaluate the effect of fecal microbiota transplantation (FMT) on the canine chronic enteropathy clinical activity index (CCECAI), fecal consistency, and microbiome of dogs with tylosin-responsive enteropathy (TRE). The trial consisted of four phases: (1) screening with discontinuation of tylosin for 4 weeks, (2) inclusion with re-introduction of tylosin for 3–7 days, (3) treatment with FMT/placebo for 4 weeks, and (4) post-treatment with follow-up for 4 weeks after treatment cessation. The study found that the treatment efficacy of FMT (71.4%) was slightly higher than that of placebo (50%), but this difference was not statistically significant due to underpowering. The most abundant bacterial species detected in the fecal microbiomes of dogs with TRE before FMT or placebo treatment were *Blautia hansenii*, *Ruminococcus gnavus*, *Escherichia coli*, *Clostridium dakarense*, *Clostridium perfringens*, *Bacteroides vulgatus*, and *Faecalimonas umbilicata*. After FMT, the microbiomes exhibited increases in *Clostridium dakarense*, *Clostridium paraputrificum*, and *Butyricicoccus pullicaecorum*. The microbiome alpha diversity of TRE dogs was lower when on tylosin treatment compared to healthy dogs, but it increased after treatment in both the FMT and placebo groups. Comparisons with the stool donor showed that, on average, 30.4% of donor strains were engrafted in FMT recipients, with the most common strains being several *Blautia* sp., *Ruminococcus gnavus*, *unclassified Lachnoclostridium*, *Collinsella intestinalis*, and *Fournierella massiliensis*.

## 1. Introduction

Treatment alternatives to antibiotics for intestinal diseases have become a high priority in research because antibiotics can cause dysbiosis of the intestinal microbiome and promote antibiotic resistance [1,2,3,4]. While some dogs show repeated improvement in gastrointestinal signs (e.g., diarrhea) after antibiotic treatment, such as in the case of tylosin-responsive enteropathy (TRE), up to 86% may relapse within a month of tylosin discontinuation [2]. This leads to repeated long-term oral application of the antibiotic with all its harmful effects on the composition of intestinal microbiota and resistance development. Consequently, there is an urgent need to find alternative treatments for TRE that do not involve antibiotics.

Fecal microbiota transplantation (FMT) is one of the most effective ways to restore the colonization of beneficial bacteria in human recipients [5,6]. The complexity and diversity of the donor microbiome may be the key factors behind the positive effects of FMT and has likely contributed to the 90% success rate in Clostridioides difficile infections (CDI) [7]. Studies in mouse and piglet models have shown a positive influence of FMT on intestinal dysbiosis [5,8,9].

The beneficial effects of FMT on microbiome dysbiosis have been demonstrated in veterinary medicine. Reports investigating the potential of FMT to tackle acute and chronic intestinal inflammation in dogs have shown positive results [10,11,12,13]. The results of a retrospective study in 41 dogs with poorly responsive chronic enteropathies (CE) suggest that FMT can be useful as an adjunctive therapy [12]. In a randomized clinical study, FMT significantly shortened hospitalization and recovery time in puppies with parvovirosis [11]. However, fecal microbiota transplantation did not show a significant positive clinical effect in a double-blinded, placebo-controlled study of 13 dogs with inflammatory bowel disease (IBD) [14]. In an interventional prospective study, the Canine Chronic Enteropathy Clinical Activity Index (CCECAI) significantly decreased in 74% (20/27) of dogs with chronic enteropathy (CE) 15 days after 1 month of treatment with daily oral freeze-dried FMT capsules [15]. However, to our knowledge, the impact of FMT in dogs with TRE has not been examined.

Here, we present a double-blinded, placebo-controlled clinical trial and proof-of-concept study to assess the efficacy of oral freeze-dried FMT on CCECAI scores, fecal consistency, and the microbiome in dogs with TRE.

## 2. Materials and Methods

### 2.1. Study Population and Ethics

Study dogs (N = 14, 7 FMT, 7 placebo) were selected from the clinical caseload visiting the Small Animal Hospital of the University of Helsinki, from referrals by private clinics, and by inviting owners who had contacted the investigators due to the advertisement of the study. All dogs were suspected of having TRE, were on tylosin treatment, and did not exhibit gastrointestinal clinical signs. The recruitment began in August 2020, and the follow-up of the last patient was completed in December 2022. The trial protocol was approved by the Finnish National Animal Experiment Board (ethical license ESAVI/43892/2020), and written informed consent was obtained from the owners of the dogs involved in the study. The trial complied with the Guidelines on Good Clinical Practices (CVMP/VICH/595/98) and Guideline on the Assessment of the Efficacy of Feed Additives, Article 7.6, of Regulation (EC) No 1831/2003.

### 2.2. Trial Design

This study was a prospective, randomized, double-blinded, placebo-controlled clinical trial on the efficacy of FMT in dogs with TRE. The trial consisted of the screening, inclusion, treatment, and post-treatment phases. At the end of each phase, there was one hospital visit, except in the inclusion phase, which included two visits (inclusion and endoscopy). Fecal samples were collected at each hospital visit. The screening phase comprised a clinical history review, physical examination, analysis of blood samples for complete blood count (CBC), serum biochemistry profile, including cobalamin, folate, and trypsin-like immunoreactivity concentrations, urinalysis, and fecal parasite examination. Results of clinical history, physical examination, and standard laboratory tests were used to assess canine chronic enteropathy clinical activity index (CCECAI). Fecal consistency scores (FCS) were assessed using a nine-point scoring system [2].

**Figure 1:**
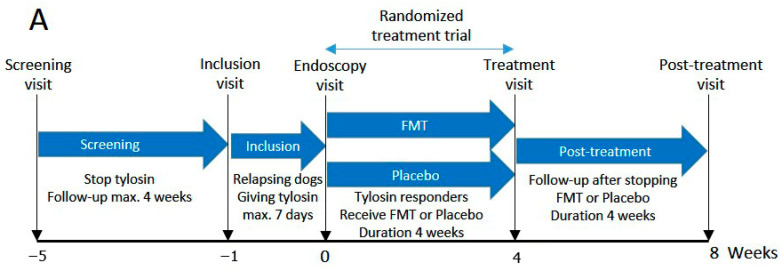

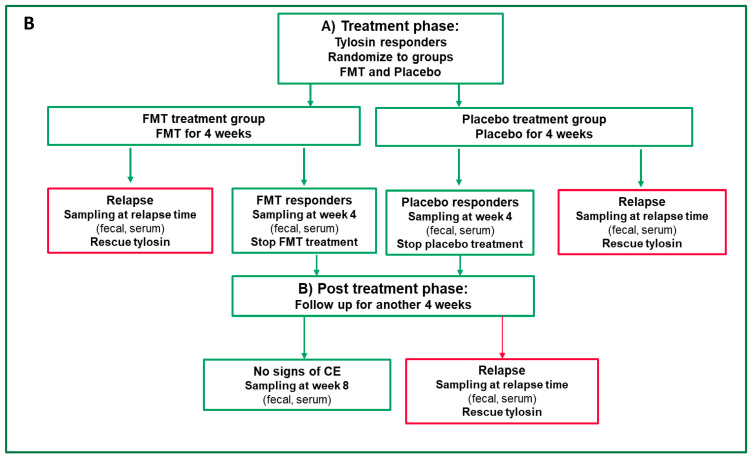
(**A**) Study design of this prospective, double-blinded, placebo-controlled clinical trial in dogs with tylosin-responsive enteropathy (TRE) receiving oral FMT or placebo capsules; (**B**) a flow chart detailing the treatment and post-treatment phases.

After the screening visit, tylosin treatment was stopped, and a maximum 4-week follow-up period was commenced to determine the recurrence of signs of CE. Dogs that relapsed after discontinuation of tylosin entered the inclusion phase (Figure 1A and Supplementary Figure S1). In this phase, tylosin (25 mg/kg q24 h) was reintroduced for 3–7 days, and dogs that responded to tylosin underwent gastroduodenoscopy/colonoscopy. Tylosin was continued during and after endoscopy for 2 more days and ceased before starting FMT or placebo treatment. Supplementary Figure S1 illustrates the design of the screening and inclusion phases in detail. During the trial, antibiotics, glucocorticoids, nonsteroidal anti-inflammatory drugs (NSAIDs), probiotics, and raw diet were not permitted. For a full list of inclusion and exclusion criteria, see Supplementary Table S1. The diet information of each dog with TRE that participated in the FMT/placebo trial is shown in Supplementary Table S11.

The treatment phase ended 4 weeks after starting FMT/placebo or at the time of relapse (Figure 1). Dogs were randomized to receive either FMT or placebo. Dogs that did not exhibit clinical signs during the treatment phase were considered responders and were transitioned to the post-treatment phase. When a dog developed clinical signs of CE within 4 weeks of the treatment phase, it was considered a non-responder and treated with tylosin. In the post-treatment phase, treatment with FMT/placebo was discontinued, and the owners monitored their dogs for another 4 weeks of follow-up (Figure 1). If no clinical signs were observed, then the dog reached the endpoint of the study.

### 2.3. Definition of Clinical Remission and Relapse

In this trial, the CCECAI and FCS were used to define clinical remission and relapse. The total CCECAI score represents the sum of nine individual scores (attitude/activity, appetite, vomiting, fecal consistency, defecation frequency, weight loss, serum albumin level, ascites and peripheral edema, and pruritus), each scored on a 0–3 scale. CCECAI was classified as insignificant (score 0–3), mild (score 4–5), moderate (score 6–8), severe (score 9–11), or very severe (score  ≥  12) [16]. As diarrhea is a major sign of CE, FCS was also assessed to determine clinical remission. For this, owners evaluated their dogs’ fecal consistency during each defecation and scored it on a 9-point scale (1 to 5, with half-point intervals) [2]. Clinical remission was defined as a CCECAI score between 0–3 and a mean FCS of three or fewer during the trial. Relapse was defined as having a CCECAI score > 3 or clinical signs of CE and FCS ≥ 4.

### 2.4. Test Product and Mode of Patient Randomization

#### 2.4.1. Fecal Microbiota Transplantation (FMT) or Placebo Products

The test product was FMT capsules obtained from AnimalBiome, Oakland, CA, USA, which contained 100% freeze-dried bacterial communities derived from the feces of a single healthy canine donor. Health was verified via veterinary records, and negative results on PCR-based parasite (Antech Diagnostics) and pathogen testing (IDEXX). These tests specifically screened for *Cryptosporidium* spp., *Campylobacter*, *Clostridioides difficile* toxins A and B, *Clostridium perfringens* α-toxin and enterotoxin A, *Salmonella* spp., *Giardia* spp., viruses (coronavirus, parvovirus, circovirus, and distemper virus), tapeworms, hookworms, roundworms, and cestodes [17]. The donor was also screened for microbiome composition and compared to a reference set of healthy dogs [18]. Furthermore, the stool donor had not been on any antibiotics in the past year, was not taking medications, and had no health conditions or infections [17].

The placebo capsules contained 100% corn starch, also obtained from AnimalBiome, Oakland, CA, USA. The visual appearance and product labels of both the placebo and FMT capsules were identical. The capsules were refrigerated at +2–+8 °C until administration.

Both the FMT and placebo capsules were administered orally. The FMT or placebo capsules were administered to dogs once a day in the morning, just before a meal. The dosages of FMT and placebo for dogs with different body weights were as follows: 5–10 kg: 1 capsule per day; 11–20 kg: 2 capsules per day; 21–30 kg: 3 capsules per day; and >30 kg: 4 capsules per day.

#### 2.4.2. Mode of Randomization

Treatment code unblinders (scratch cards) that described the study dog’s treatment were provided by Orion Corporation, Espoo, Finland, to the investigator if a dog needed to be unblinded due to an adverse event. The treatment code was opened after locking the study database.

In the screening phase, the dogs received study numbers in the order they were enrolled in the study. At the start of the treatment phase, the investigator provided numbered capsule bottles to each owner in ascending order, starting from No. 1. This randomization procedure ensured that half of the dogs were on FMT, and the other half were on placebo.

### 2.5. Endoscopy and Histopathology

The study participants underwent gastrointestinal endoscopy before entering the treatment phase. Endoscopic biopsies were collected from the stomach, duodenum, ileum, and colon, fixed in 10% buffered formalin, embedded, sectioned at 4 µm, and stained with hematoxylin and eosin before examination and scoring according to the WSAVA guidelines by a board-certified pathologist.

### 2.6. Fecal Dry Matter

Fecal dry matter (FDM) percentage was measured from fecal samples using a previously described method [19]. Briefly, frozen fecal samples (−80 °C) were thawed overnight at +4 °C, then ~0.5 g of fecal sample was uniformly distributed on an aluminum plate and placed in a moisture meter device (MB-25; Ohaus Co., Ltd., Parsippany, NJ, USA). The feces were dried at 120 °C and when the weight needed to show a reduction of less than 1 mg after heating for 60 s. After the heating process was complete, the dry weight was measured. FMD (%) was calculated as follows: ((moisture weight − dry weight)/moisture weight) × 100%. FDM measurement was performed twice for each fecal sample, and the mean percentage was reported.

### 2.7. Healthy Dog Microbiomes

In addition to collecting fecal samples from study participants, we also collected fecal samples from 30 apparently healthy dogs to serve as a reference. These dogs were all located in Finland. At the time of the sampling, the dogs did not show any signs of gastrointestinal distress or other clinical symptoms, and they had not undergone any type of antibiotic therapy for at least 3 months before the sampling collection time. They were free of internal and external parasites. Of the 30 dogs, 12 (40%) were female and 18 (60%) were male. The median age was 4 years old, and the median weight was 15.5 kg. Detailed information regarding this population is provided in Supplementary Table S2. Fecal samples were collected by the owners and placed into 2 mL screw cap tubes containing 70% ethanol. The bacterial composition of samples stored in ethanol closely resembles that of fresh samples [20]. We opted for this storage and preservation method to minimize the number of freeze-thaw cycles. These tubes were shipped to AnimalBiome facilities (Oakland, CA, USA) and stored at 4 °C until further laboratory analysis. Supplementary Table S2 summarizes the demographic characteristics of these healthy dogs.

### 2.8. DNA Extraction and Full-Length 16S rRNA Amplicon Gene Sequencing of Fecal Microbiome

To gain insight into the fecal microbiomes of canine participants and healthy dogs, genomic DNA was extracted from fecal samples using the QIAGEN DNeasy PowerSoil Pro HT kit (QIAGEN, Germantown, MD, USA), following the manufacturer’s instructions. The DNA concentration was measured using a Qubit dsDNA High-Sensitivity Kit (ThermoFisher, Waltham, MA, USA). Primers 27F (50-AGRGTTYGATYMTGGCTCAG-30) and 1492R (50-RGYTACCTTGTTACGACTT-30), tailed with 16 bp asymmetric barcode sequences, were used for full-length (V1 to V9) 16S rRNA gene amplification. PCR amplification was performed using 12.5 μL of KAPA HiFi HotStart ReadyMix PCR kit (KAPA Biosystems, Wilmington, MA, USA), 3 μL of both forward and reverse primers (2.5 μM), 5 μL of template DNA, and PCR-grade water required for a final volume of 25 μL. PCR conditions were as follows: initial denaturation at 95 °C for 3 min, followed by 25 cycles of denaturation at 95 °C for 30 s, annealing at 57 °C for 30 s, and extension at 72 °C for 60 s. Purified amplicons were sequenced using PacBio Sequel IIe chemistry (Pacific Biosciences, Menlo Park, CA, USA).

### 2.9. Bioinformatic Processing and Data Analysis of Fecal Microbiota Sequencing

Following sequencing, circular consensus sequencing reads (CCS) were converted to HiFi reads for each demultiplexed sample using the Single Molecule Real-Time (SMRT) Link Application software (v.11.0.0.146107). These HiFi reads were then processed through Quantitative Insights Into Microbial Ecology (QIIME2, version 2021.8) software [21] and the Divisive Amplicon Denoising Algorithm (DADA2, v1.14.1) plugin [22] for trimming, denoising, dereplication, and chimera filtration. Taxonomic assignment of Amplicon Sequence Variants (ASVs) was primarily performed using a Naive Bayes trained sklearn classifier in QIIME2, with a confidence threshold of 0.7, and further refined using VSEARCH classification [23]. The Silva (v.138.1 NR99) reference database [24] was manually curated to exclude sequences that did not meet specific criteria, significantly reducing its size and enhancing the specificity of taxonomic labels. For detailed processing steps and criteria, refer to AnimalBiome’s tutorial on processing PacBio full-length 16S rRNA HiFi reads (https://github.com/AnimalBiome/AB_FlexTax/tree/main, accessed on 2 June 2024).

### 2.10. Statistical Analysis

#### 2.10.1. Analysis of CCECAI, FCS, and FDM Variables

The aim of this study was to assess the efficacy of FMT in treating dogs with tylosin-responsive enteropathy compared to placebo by examining CCECAI scores and fecal dry matter percentage. Primary statistical analysis was performed with the full analysis set of animals (all randomized subjects). In the following circumstances, randomized subjects were excluded from the full analysis set: (1) failure to satisfy major entry criteria (eligibility violations); (2) failure to take at least one dose of trial medication; and (3) lack of any data post-randomization (lack of data from examination at the end of treatment/post-treatment phases due to discontinuation). The proportion of CCECAI score, as a primary efficacy variable, was analyzed in FMT/placebo responder dogs at the end of treatment (primary endpoint) and post-treatment (secondary endpoint) phases using a logistic regression model. In addition, the change in CCECAI from baseline (before starting FMT/placebo treatment, endoscopy visit) to the end of treatment and post-treatment phases was analyzed using an analysis of covariance model, including the baseline CCECAI score as a covariate. The secondary efficacy variable, the fecal consistency mean score, was evaluated using the same analysis methods as for the primary efficacy variable. In addition, the change from baseline was also analyzed for the other selected efficacy variables using an analysis of covariance model, including the baseline value as a covariate. To predict fecal consistency scores based on fecal dry matter percentage, a simple linear regression test was used.

The data are presented as the mean (standard deviation) or median (range), as appropriate. For all analyses, we considered values of *p* < 0.05 as significant. All statistical analyses were performed using SAS for Windows version 9.4 (SAS Institute Inc., Cary, NC, USA).

#### 2.10.2. Microbiome Statistical Analyses

Microbiome data were imported into the R statistical software program (v.4.3.0) [25] for subsequent analysis. Plots of microbiome composition showcasing the relative abundances of bacterial genera or species were done with the ggplot package (v.3.4.2). Differential abundance analysis was performed using the LinDA package (v.0.1.0) [26]. The LinDA model was designed to include the sample type (before FMT versus after FMT treatment, or before placebo versus after placebo treatment) as the primary predictor. Additionally, the model accounted for repeated measures by incorporating dog ID as a random effect to account for repeated measures in the same individual. The prevalence cutoff was set at 30%, the winsorization cut-off (quantile) at 0.97, and *p*-value adjustment at “FDR”.

Because we had microbiome information on both the stool donor and FMT recipients, we examined the bacterial engraftment. Bacterial engraftment rates were calculated by dividing the number of amplicon sequence variants (ASVs) detected in both postFMT samples (from the treatment and post-treatment phases) and stool donor samples (excluding ASVs shared between pre-FMT [i.e., endoscopy] samples and donors) by the total number of ASVs in the donor sample (again, excluding ASVs shared between endoscopy and donor samples), as was done by Rojas et al. (2024) [27]. Before calculating these rates, we removed singleton and doubleton ASVs (ASVs with a summed count of 1 or 2 reads in the entire dataset), as these could otherwise inflate ASV engraftment rates.

For alpha- and beta-diversity analyses, the data were further processed using the R package MicrobiomeAnalystR (Xia Lab, McGill University, Montreal, QC, Canada) [28]. A low-count filter was used to filter all features with <4 counts in at least 20% of the values. Features with <10% variance, based on the inter-quartile rank, were filtered using a low-variance filter. Finally, for data scaling, the total sum scaling was applied at the bacterial genus level. Alpha-diversity analyses were performed using the Shannon index and Kruskal–Wallis nonparametric test, whereas beta-diversity analyses were performed using the Bray–Curtis dissimilarity index and PER-MANOVA tests [29].

## 3. Results

### 3.1. Study Population

Fourteen dogs participated in this study and received oral FMT (n = 7) or placebo (n = 7) capsules for a maximum duration of 4 weeks. They had a median age of 2.5 years, a median body weight of 18.9 kg, and 50% were neutered (Table 1). There were slightly more males (57.1%) than females (42.9%), and the most common breeds were Collie (n = 4) and Cavalier King Charles Spaniel (n = 2).

However, to arrive at these numbers, 55 owners regarded their dogs as suspected of having TRE and allowed their recruitment for this study. In 29/55 dogs, suspected TRE was confirmed, and they advanced to the screening examination (Supplementary Figure S2). Then, tylosin treatment was stopped and was followed by a maximum 4-week period to assess relapse and recurrence of signs consistent with CE. Also, 12/29 dogs were excluded at this stage because they did not show signs of CE or had concurrent diseases that prevented further inclusion. The remaining dogs (n = 17) entered the inclusion phase. Of these, 14 responded to tylosin within 3–7 days, underwent endoscopy (with the exception of 2 dogs), and entered the treatment phase. Tylosin treatment continued for 2 more days after the endoscopy, and on the 3rd day, tylosin was discontinued, and the 4-week FMT, or placebo treatment, began.

### 3.2. Outcomes Based on CCECAI Scores

The CCECAI was assessed at the screening, inclusion, endoscopy, end of treatment (primary endpoint), and post-treatment (secondary endpoint) phases. CCECAI was scored at the time of relapse for dogs that did not reach all study endpoints.

Of the 14 dogs entering the treatment phase, one dog from the placebo group was excluded from the study because of pyometra. According to the CCECAI scores, two dogs from the FMT group (2/7, 28.6%) and three dogs from the placebo group (3/6, 50%) relapsed during the treatment phase. Thus, a total of eight dogs (5 FMT, 3 placebo) proceeded to the post-treatment phase. However, one dog from the FMT group was excluded because it received corticosteroids for a skin condition. In the post-treatment follow-up phase, two more dogs in the FMT group relapsed, bringing the total number of relapsed dogs to 66.7%. None of the patients in the placebo group experienced relapse. There was no significant difference between the FMT and placebo groups in terms of relapse rates at the end of the treatment (*p* = 0.433) or post-treatment (*p* = 0.56) phases.

**Figure 2:**
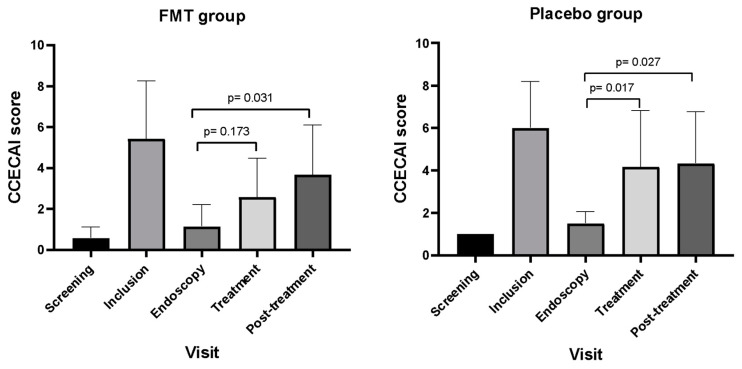
Mean (SD) of CCECAI score during different visits of dogs in FMT and placebo groups. Data are expressed as the mean  ±  standard deviation.

Furthermore, compared to the endoscopy visit as a baseline value, the mean (SD) CCECAI score of the FMT group was not significantly different [2.6 (1.9) vs. 1.1 (1.1), *p* = 0.173] at the treatment visit (Figure 2). However, dogs that received a placebo had a significantly higher mean CCECAI score [4.2 (2.6) versus 1.5 (0.5), *p* = 0.031] at the treatment visit. In the post-treatment visit, mean CCECAI scores in both FMT [3.7 (2.4) versus 1.1 (1.1), *p* = 0.017] and placebo [4.3 (2.4) versus 1.1 (1.1), *p* = 0.027] groups significantly increased compared to the endoscopy visit. Calculations of mean CCECAI scores in the post-treatment visit included the CCECAI scores of dogs that relapsed during the treatment phase.

### 3.3. Outcomes Based on Fecal Consistency Scores

Fecal consistency scores (FCS) were assessed at screening, inclusion, endoscopy, end of treatment (primary endpoint), and post-treatment (secondary endpoint) visits in dogs reaching those endpoints. In dogs that did not reach the endpoints, FCS was scored at the time of relapse.

Of the 14 dogs entering the treatment phase, one dog was excluded from the placebo group because of pyometra. In the FMT group, one dog in the treatment phase was excluded from the group because the treatment visit data (4 weeks after starting FMT treatment) were not available as the relapse occurred earlier. The dog relapsed based on the CCECAI score but had a normal FCS. The dog was not considered a responder based on the FCS of a single day, since the protocol defines the responder as having a mean FCS score ≤3 during the last 3 days of treatment or post-treatment phases. However, the dogs’ FCS score was used for the CCECAI calculation on the day of relapse. Based on the FCS, one dog from the FMT group (1/6, 16.7%) and three dogs from the placebo group (3/6, 50%) relapsed in the treatment phase.

Eight dogs continued to the post-treatment phase. However, from the FMT group, two dogs were excluded: one due to receiving corticosteroids and another due to not having post-treatment visit data available since the relapse occurred earlier. Based on the FCS, one more dog relapsed during the post-treatment phase in the FMT group. Therefore, 2/4 dogs (50%) did not reach the second endpoint and were considered non-responders. In the placebo group, none of the three dogs relapsed during the post-treatment phase. Therefore, the relapse rate stayed at 3/6 (50%). There was no significant difference between the FMT and placebo groups in terms of relapse rates at the end of the treatment (*p* = 0.23) or post-treatment (*p* = 1) phases.

**Figure 3:**
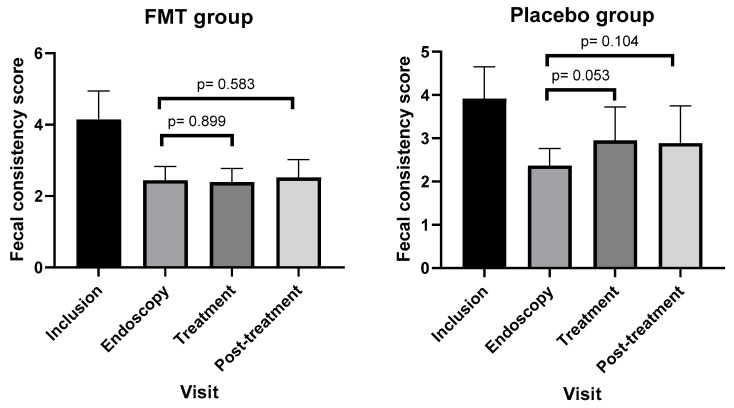
Mean (SD) of fecal consistency score (FCS) in different visits of dogs in FMT and placebo groups. Data are expressed as the mean  ±  standard deviation.

Compared to the endoscopy visit as a baseline value, the mean FCS score of the FMT group did not change significantly [2.39 (0.39) versus 2.44 (0.38), *p* = 0.899] in the treatment visit (Figure 3). However, dogs that received a placebo had a higher score with a trend toward statistical significance [2.95 (0.77) versus 2.37 (0.4), *p* = 0.053]. In the post-treatment visit, the mean FCS score in both the FMT [2.52 (0.50) versus 2.44 (0.38), *p* = 0.583] and placebo [2.88 (0.87) versus 2.37 (0.4), *p* = 0.104] group did not change significantly compared to the endoscopy visit (Figure 3). For the calculation of the mean FCS score in the post-treatment visit, FCS scores of dogs that relapsed in the treatment phase were considered.

### 3.4. Fecal Dry Matter

**Figure 4:**
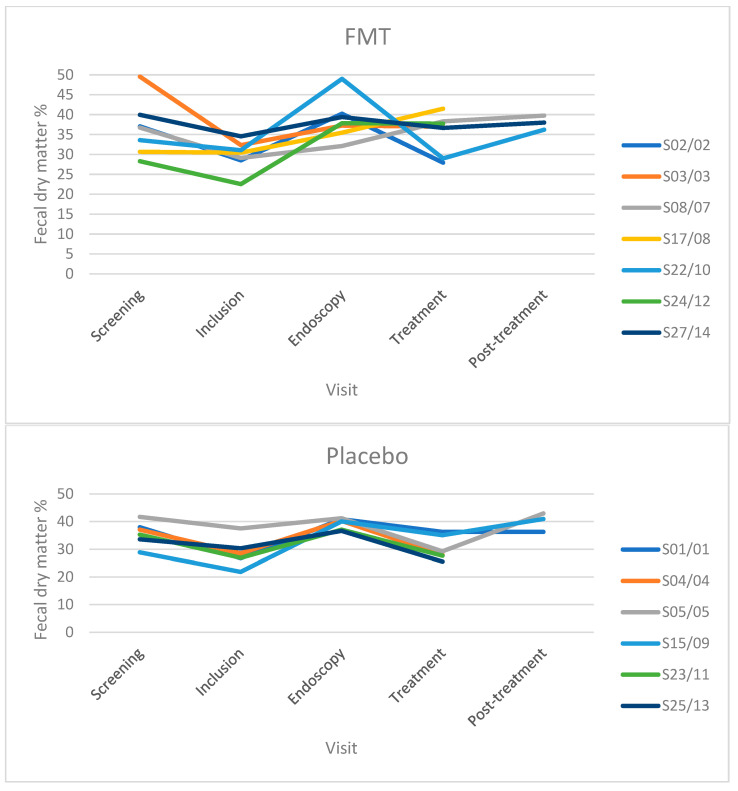
Fecal dry matter percentage in individual dogs of both FMT (**top**) and placebo groups (**bottom**) throughout the study.

**Figure 5:**
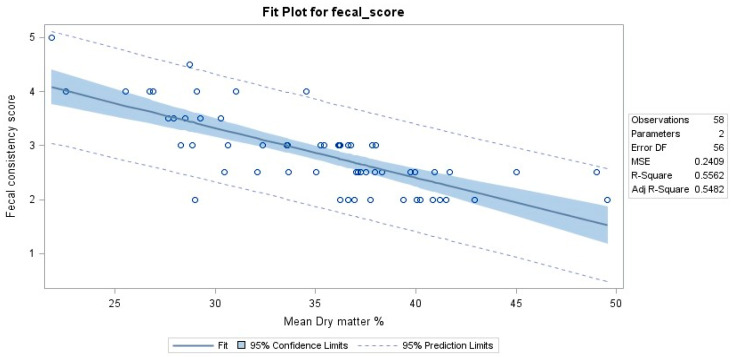
Linear regression analysis correlating fecal consistency scores and dry matter percentage.

Fecal dry matter (FDM) percentage was measured in TRE dogs from both the FMT and placebo groups. However, due to the low number of dogs in different visits, the data are reported descriptively (Table 2 and Figure 4). The data of the two FDM measurements, their mean values, standard deviations, and coefficient of variation (%) are shown in Supplementary Table S12. The results showed that the median FDM% decreased from screening to inclusion in both the FMT and placebo groups when the included dogs showed a relapse of clinical signs, including diarrhea. FDM% increased from inclusion to endoscopy in both groups when all dogs received tylosin for the second time and responded with improvement in clinical signs. In the FMT group, FDM% was greater during the treatment and post-treatment visits than at the inclusion visit in dogs that did and did not relapse. For the placebo group, FDM% at the end of treatment and post-treatment visits remained higher than that at the inclusion visit in dogs that did not relapse but decreased in dogs that relapsed (Table 2). Results from linear regression indicated that FMD% accounted for 56% of the variation in fecal scores. Results from a linear regression indicated that FMD% accounted for 56% of the variation in fecal scores. A strong negative correlation [correlation coefficient r = −0.75] between the two variables is apparent in the linear regression plot (Figure 5).

### 3.5. The Composition of Fecal Microbiomes before and after Treatment

**Figure 6:**
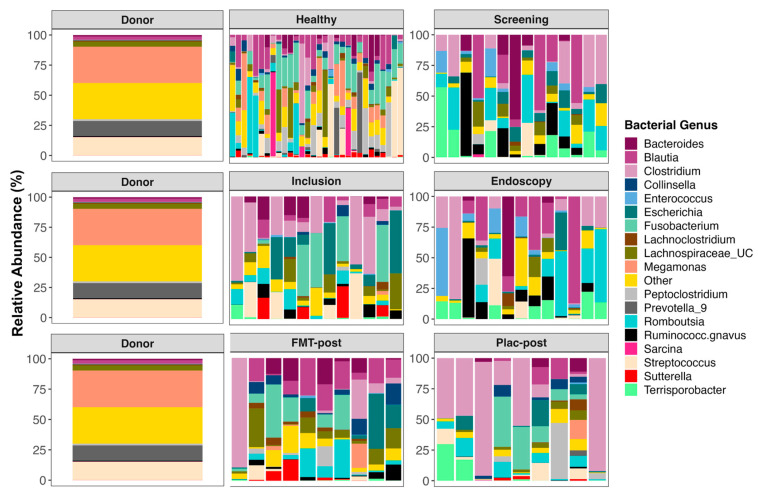
Fecal microbiome composition (genus level) of the stool donor, healthy dogs, and dogs receiving FMT or placebo treatment. The relative abundances of bacterial genera with mean relative abundances of less than 1% are shown; all others are combined into the category “Other”. Samples are grouped by time point as follows: screening, inclusion, and endoscopy. “Post” samples are from the treatment and post-treatment visits. Plac—placebo.

Prior to starting FMT or placebo treatment, the fecal microbiomes of TRE dogs were dominated by *Clostridium*, *Romboutsia*, *Terrisporobacter*, *Enterococcus*, *Streptococcus*, *Blautia*, *Escherichia*, *Fusobacterium*, *Ruminococcus*, unclassified *Lachnospiraceae*, and *Bacteroides* (Figure 6, for average relative abundances of bacterial genera by group; see Supplementary Table S3). After FMT treatment, canine participants also harbored significant abundances of the same taxa, but also of *Collinsella* (5.46% mean relative abundance), *Turicibacter* (2.77%), *Peptoclostridium* (2.52%), *Megamonas* (2.39%), and *Lachnoclostridium* (2.31%). In the placebo group, over 60% of their microbiome post-treatment was represented by three bacterial genera, *Clostridium* (40.9%), *Fusobacterium* (11.3%), and *Peptoclostridium* (6.27%). For plots of microbiome composition at each timepoint faceted by the individual, see Supplementary Figure S3.

The donor’s microbiome harbored large abundances of *Megamonas* (30.15%), *Streptococcus* (15.06%), *Prevotella* 9 (12.43%), unclassified *Bacteroidales* (5.81%), and unclassified *Lachnospiraceae* (4.26%) (Figure 6 and Supplementary Table S3). The fecal microbiomes of healthy dogs mostly contained *Fusobacterium* (14.93%), *Blautia* (9.91%), *Streptococcus* (8.7%), *Megamonas* (8.6%), *Peptoclostridium* (8.3%), *Romboutsia* (7.3%), unclassified *Lachnospiraceae* (6.18%), and *Clostridium* (5.4%) (Figure 6 and Supplementary Table S3).

**Figure 7:**
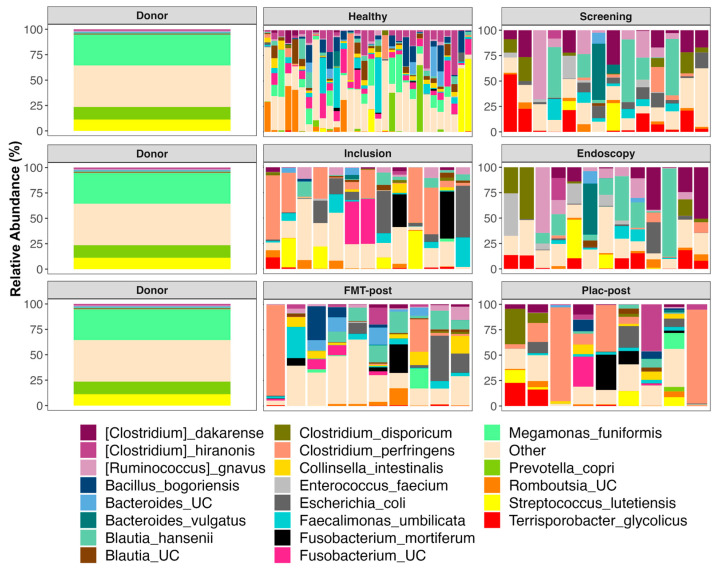
Fecal microbiome composition (species level) of the stool donor, healthy dogs, and dogs receiving FMT or placebo treatment. The relative abundances of bacterial species with mean relative abundances of less than 1.4% are shown; all others are combined into the category “Other”. Samples are grouped by time point as follows: screening, inclusion, and endoscopy. “Post” samples are from the treatment and post-treatment visits. Plac—placebo.

When examining the relative abundances of bacterial species, the results showed that the most abundant taxa in fecal microbiomes from TRE dogs before FMT or placebo treatment were: *Blautia hansenii*, *Ruminococcus gnavus*, *Escherichia coli*, *Clostridium dakarense*, *Clostridium perfringens*, *Bacteroides vulgatus*, *Faecalimonas umbilicata*, and *Clostridium disporicum* (Figure 7, for average relative abundances of bacterial species by group; see Supplementary Table S4). TRE dogs from the placebo group, in particular, also harbored significant abundances of *Streptococcus lutetiensis* (7.7%), *Enterococcus faecium* (5.54%), and *Fusobacterium mortiferum* (2.43%). After FMT, the microbiomes shifted and contained more *Collinsella intestinalis* (5.48%), *Bacillus bogoriensis* (4.63%), *Fusobacterium mortiferum* (3.98%), and *Romboutsia ilealis* (3.91%) (Figure 7 and Supplementary Table S4). The fecal microbiomes of dogs’ post-placebo treatment contained the same dominant taxa as before the treatment began. For plots of bacterial species composition at each timepoint faceted by the individual, see Supplementary Figure S4.

For the stool donor, 60% of its fecal microbiome was composed of *Megamonas funiformis* (30.15%), *Prevotella copri* (12.43%), *Streptococcus lutetiensis* (11.13%), and unclassified *Bacteroidales* (5.81%) (Figure 7, Supplementary Table S4). Meanwhile, the fecal microbiomes of healthy dogs mostly consisted of *Megamonas funiformis* (8.63%), *Clostridium hiranonis* (8.35%), *Bacillus bogoriensis* (6.6%), *Faecalimonas umbilicata* (6.49%), *Streptococcus lutetiensis* (5.37%), and *Prevotella copri* (4%) (Figure 7 and Supplementary Table S4).

**Figure 8:**
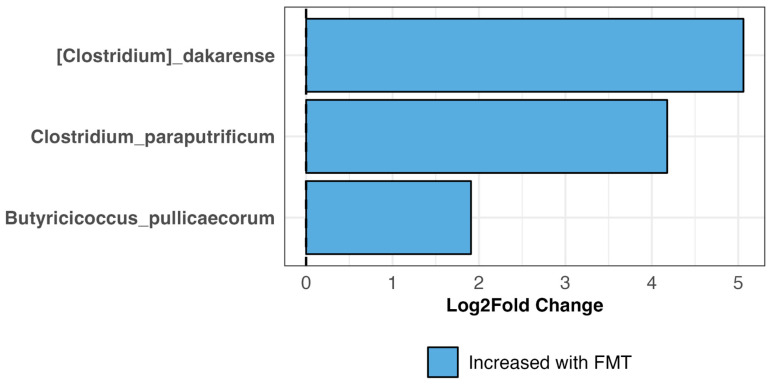
Bacterial species that significantly increased in TRE dogs receiving FMT treatment according to differential abundance analysis with LinDA. None of the bacterial species examined significantly increased or decreased in TRE dogs receiving the placebo.

To statistically determine which bacterial species increased or decreased in the FMT recipients compared to the placebo group, we performed differential abundance analysis. The results showed that the relative abundances of three bacterial species, *Clostridium dakarense*, *Clostridium paraputrificum*, and *Butyricicoccus pullicaecorum,* increased significantly in TRE dogs receiving FMT treatment (LinDA *p* = 0.04, *p* = 0.05, *p* = 0.08) (Figure 8). No bacterial species significantly increased or decreased as a result of placebo treatment (LinDA *p* > 0.1).

### 3.6. Fecal Microbiome Alpha- and Beta-Diversity before and after Treatment

**Figure 9:**
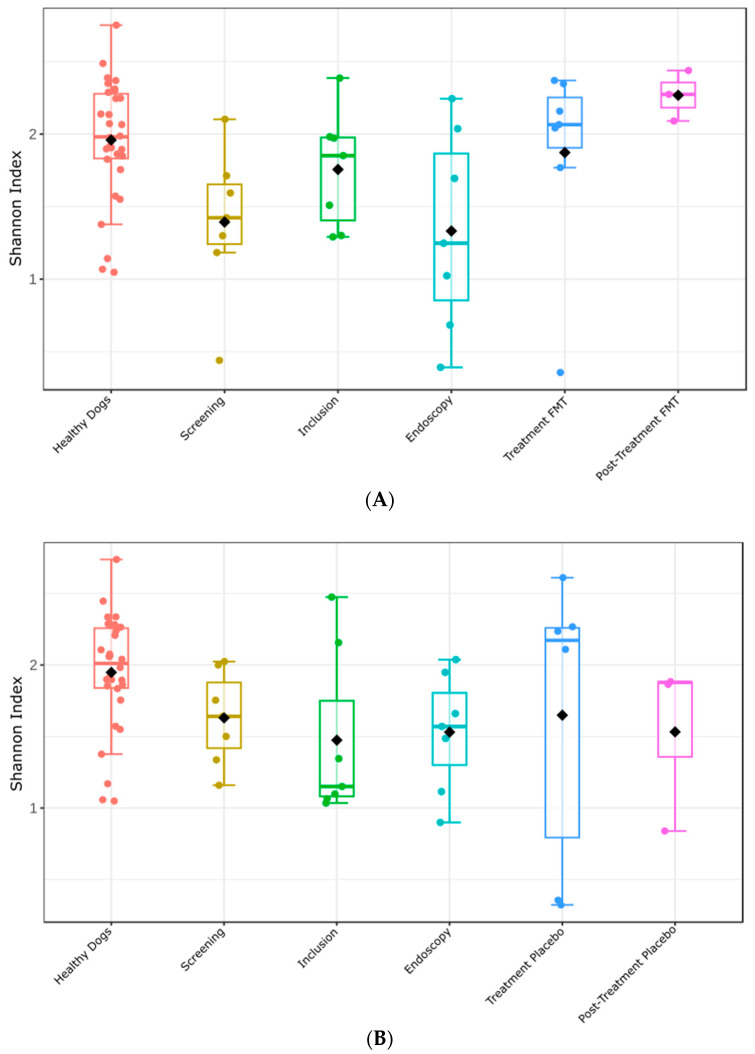
Microbiome alpha diversity (Shannon index) among TRE dogs in the FMT (**A**) and placebo (**B**) clinical groups at different time points compared to healthy dogs. The horizontal line inside each box plot represents the median; the top and bottom of each box represent the 75th and 25th percentiles, respectively; and the whiskers represent the 95th and 5th percentiles. Black squares represent mean values.

Next, we investigated whether fecal microbiome alpha diversity differed between healthy dogs and TRE dogs at various treatment phases (e.g., screening, inclusion, endoscopy, etc.). The fecal microbiome alpha diversity (Shannon index) of dogs from the FMT treatment group at the screening and endoscopy visits was significantly lower than that of healthy dogs (Figure 9A) (Kruskal–Wallis Shannon index: screening versus healthy dogs, *p* = 0.010; endoscopy versus healthy dogs, *p* = 0.019). At the inclusion visit, fecal microbiome alpha diversity did not differ between the two groups (Kruska–Wallis Shannon index, *p* = 0.259). Similar to the TRE dogs from the FMT group, dogs from the placebo group had significantly lower fecal microbiome alpha diversity at the screening and endoscopy visits than healthy dogs (Figure 9B) (Kruskal–Wallis Shannon index: screening versus healthy dogs, *p* = 0.036; endoscopy versus healthy dogs, *p* = 0.021). At the inclusion visit, no significant differences in microbiome alpha diversity were detected between the two groups (*p* = 0.071).

After receiving FMT in the treatment visit, the alpha diversity of the fecal microbiome in TRE dogs increased compared to the screening visit, although the *p*-value hovered around significance (Shannon index: treatment visit versus screening visit, *p* = 0.073), but the same trend was not observed between dogs at the endoscopy visit and at the treatment visit (treatment visit versus endoscopy visit, *p* = 0.128). Overall, the alpha diversity kept increasing over time, showing a significant difference between dogs at the screening and endoscopy visits compared to dogs at the post-treatment visit (post-treatment visit versus screening visit, *p* = 0.033; post-treatment visit versus endoscopy visit, *p* = 0.033). Overall, it can be concluded that alpha diversity increased also in dogs receiving placebo treatment. However, the differences between dogs at different time points were not significant. The pairwise comparisons of the Shannon Index between all time points in the FMT and placebo groups and the healthy dogs are shown in Supplementary Tables S5 and S6.

**Figure 10:**
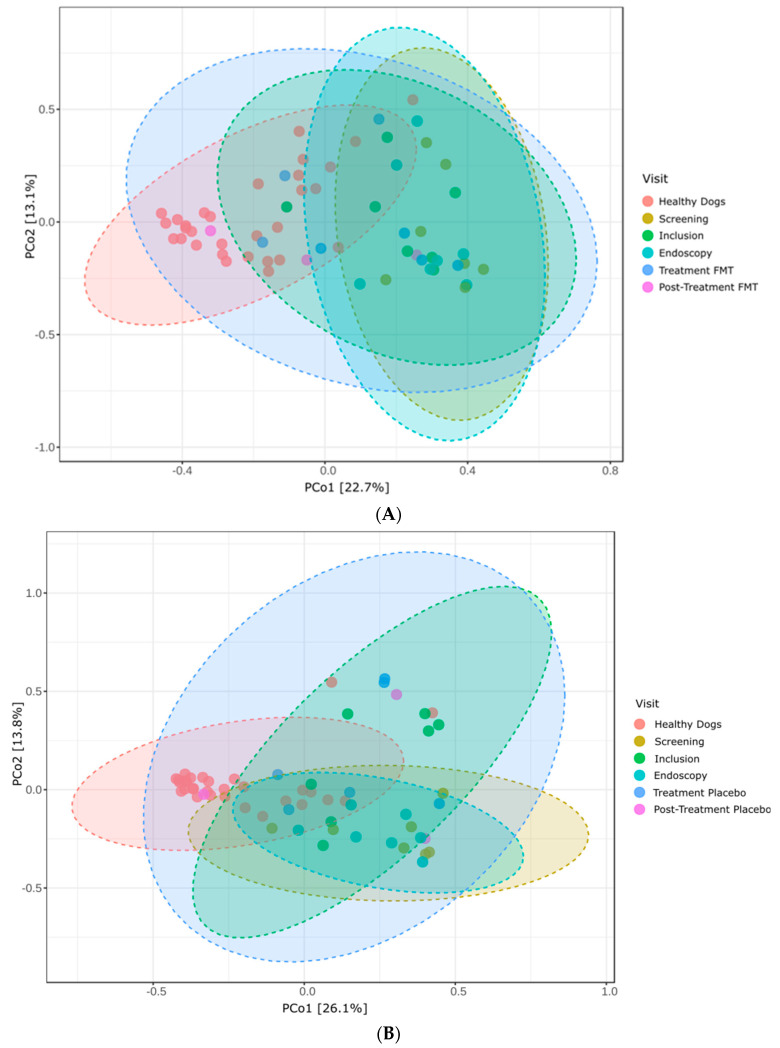
Beta diversity as assessed by the Bray–Curtis dissimilarity index, illustrating similarities and differences in community composition. (**A**) Comparison between all visits for the FMT group and healthy dog group. PERMANOVA *p*-value 0.001. (**B**) Comparison between all visits for the placebo group and healthy dog group. PERMANOVA *p*-value 0.001.

Furthermore, we analyzed the degree of similarity or dissimilarity of the bacterial communities among the groups. Beta diversity, as assessed using the Bray–Curtis dissimilarity index, revealed a notable level of diversity among all cohorts, thereby corroborating the findings delineated by the Shannon index. PERMANOVA analysis further substantiated this diversity, yielding a statistically significant *p*-value of 0.001 (Figure 10). Dogs that received FMT treatment were statistically different from healthy dogs at all visit points, in addition to the post-treatment visit. Moreover, there was a statistically significant difference between dogs at the inclusion visit and dogs at the endoscopy visit (Supplementary Table S7). Dogs that received the placebo treatment were statistically different from the healthy dogs at all visit time points, except the post-treatment visit, although in this case, this comparison showed a trend (*p* = 0.05). In addition, beta diversity was significantly different between dogs at the screening and treatment visits (*p* = 0.04), at the endoscopy visit compared to the treatment visit (*p* = 0.008), and at the inclusion visit compared to the endoscopy visit (*p* = 0.045) (Supplementary Table S8).

### 3.7. Bacterial Engraftment in TRE Dogs Receiving FMT Treatment

**Figure 11:**
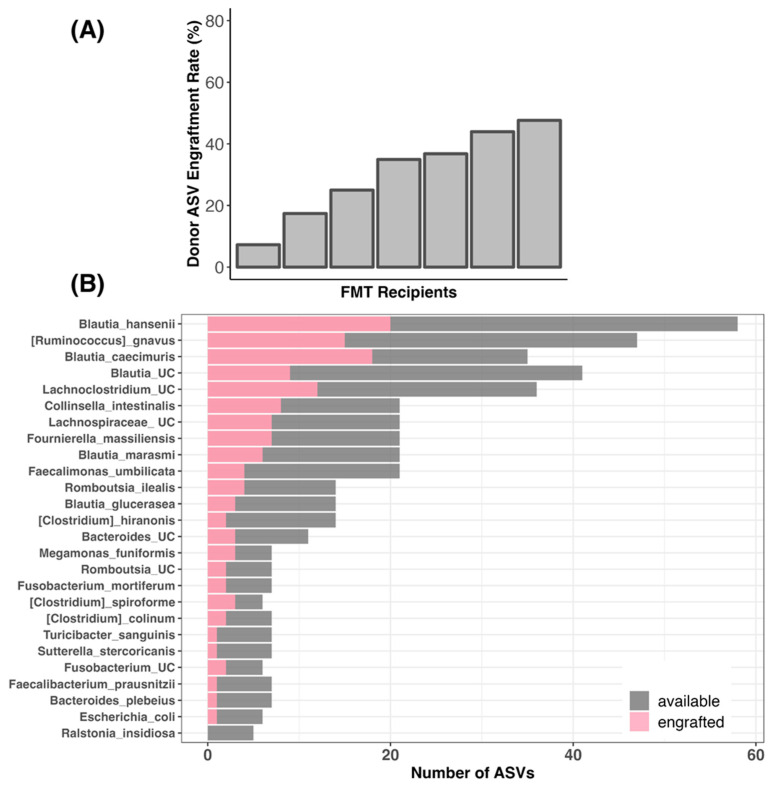
Engraftment of donor bacteria in TRE dogs receiving FMT treatment. We compared the presence of bacterial amplicon sequence variants (ASVs) found in the donor compared to FMT recipients before and after FMT treatment (excluding ASVs shared between donors and recipients pre-FMT). (**A**) Plots of donor bacterial amplicon sequence variant (ASV) engraftment rates across FMT recipients; 100% engraftment would indicate that all of the donor ASVs that could be shared were shared. (**B**) Taxonomic assignments of the donor ASVs that were most frequently shared with FMT recipients.

Lastly, because we had information on the fecal microbiome profiles of both FMT recipients and stool donor, we compared the degree to which donor bacteria were “engrafted” in FMT recipients. We analyzed the bacterial amplicon sequence variants (ASVs), which reflect the most refined level of taxonomy, found in the donor’s microbiome and in the FMT recipients’ microbiome at the endoscopy or treatment/post-treatment phases. We discovered that 7.24% to 47.61% of the stool donor’s ASVs were engrafted in the FMT recipients, with an average of 30.41% and a median of 34.92% (Figure 11A and Supplementary Table S9). This indicates that, out of the 56–69 bacterial ASVs present in the donors with the capacity to engraft, about 30.4% on average (5–30 ASVs) were successfully engrafted in the FMT recipients (Supplementary Table S9). The most commonly engrafted ASVs were classified as *Blautia hansenii*, *Ruminococcus gnavus*, *Blautia caecimuris*, unclassified *Blautia*, unclassified *Lachnoclostridium*, *Collinsella intestinalis*, *Fournierella massiliensis*, and *Blautia marasmi* (Figure 11B). Bacterial taxa such as unclassified *Romboutsia*, *Clostridium colinum*, and *Faecalibacterium prausnitzii* did not engraft as well (Figure 11B).

### 3.8. Adverse Events (AEs)

Short-term mild-to-moderate AEs occurred in 2/7 dogs from the FMT group and 5/6 dogs from the placebo group during the treatment phase. They were unrelated to FMT or placebo treatment and included short-term diarrhea, urinary incontinence, bleeding during colonoscopy, intubation-related coughing, perianal fistula, and hematuria. During the post-treatment phase, one dog in the FMT group was excluded and two dogs relapsed. The excluded dog and one of the relapsed dogs had unexpected severe AEs at 11 and 16 days after discontinuing FMT, respectively. They were clinically diagnosed with acute pancreatitis and cholangitis. The owners of both dogs decided to euthanize, but only the dog with cholangitis was allowed to undergo necropsy, and bacterial fibro-necrotizing cholangitis was diagnosed. To rule out a causal relationship between FMT and fibronecrotic cholangitis, we cultured fecal samples from the screening, inclusion, endoscopy, treatment, and post-treatment visits, as well as from the remaining FMT capsules, and looked for Extended Spectrum Beta-Lactamase (ESBL, includes *E. coli*), *Salmonella*, *Yersinia*, and *Campylobacter*. None of the fecal samples tested positive for cultured bacteria.

### 3.9. Histopathology

Of the 14 dogs entering the treatment phase, 12 dogs underwent gastroduodenoscopy/colonoscopy (5 in the FMT group and 7 in the placebo group). However, one dog from the placebo group was excluded from the dataset due to pyometra. Based on histopathological examination, malignant changes in the gastrointestinal tract were ruled out. The total histopathological changes were calculated by accumulating individual scores, and the observed changes were classified as insignificant or mild in all the biopsied dogs (Supplementary Table S10).

## 4. Discussion

In this placebo-controlled, randomized clinical trial, FMT or placebo capsules were administered orally to dogs with TRE in order to assess the efficacy of freeze-dried FMT originating from a single healthy donor dog. To the best of our knowledge, this is the first study to examine the clinical effects of FMT in dogs with TRE. Specifically, impacts on CCECAI, FCS, FDM, microbiome composition, alpha diversity, and beta diversity were investigated.

### 4.1. Efficacy of FMT Based on CCECAI, FCS, and FDM

Our results showed that based on CCECAI scores, 71.4% (5/7) of dogs receiving oral FMT capsules and 50% (3/6) of dogs receiving placebo capsules did not relapse during the 4 weeks of treatment, although this difference was not statistically significant, potentially due to the low number of included dogs, leading to an underpowered study. Based on the power analysis, the number of dogs per group should have been 15, but due to the COVID-19 pandemic and associated shutdowns, it was impossible to achieve those recruitment numbers during the trial period. Another challenge was the study dropout rate; only 14/55 dogs (25%) suspected of having TRE were confirmed to have had TRE and entered the study. Kilpinen et al. (2011) report almost a similar drop-out rate for dogs being wrongly diagnosed with TRE [2]. Another aspect of failing to include more dogs is a recent increase in the variety of clinical diets on the veterinary market that are effective in improving chronic diarrhea and leading, therefore, to a possible decreased use of antibiotics for treating canine CE [30,31,32,33,34].

When comparing the different visits within the FMT group, the CCECAI score did not change significantly after FMT treatment compared with the endoscopy visit (on tylosin and without CE signs). However, dogs with TRE who received placebo treatment had significantly higher CCECAI scores at the treatment visit than at the endoscopy visit. This result is similar to that of Collier et al. (2022), who did not find a significant difference between FMT and placebo treatment in 13 dogs with inflammatory bowel disease (IBD), although there was a trend for dogs receiving FMT to have reductions in their disease severity, as assessed by the CCECAI at Day 30 when compared to baseline, whereas dogs receiving placebo did not [14]. However, the FMT/placebo application route in this study was as an enema, in addition to a standard treatment that included a corticosteroid and a hypoallergenic diet. In a retrospective study by Toresson et al. (2023), 41 dogs with CE did not respond satisfactorily to an elimination diet, probiotic, and/or immunosuppressive treatment. They were treated with 1–5 (median, 3) FMTs as a rectal enema, and the canine inflammatory bowel disease activity index (CIBDAI) score was compared at baseline versus 1–3 weeks after the last FMT [12]. Their results showed that the CIBDAI significantly decreased from baseline with a score of 2–17 (median 6) to 1–9 (median 2; *p* < 0.0001) after FMT treatment and concluded that the FMT enema can be useful as an adjunctive therapy in dogs with poorly responsive CE. In our study, the CCECAI score remained low when we stopped tylosin and administered FMT capsules for a maximum of 4 weeks, but the index increased significantly in the placebo group. However, after discontinuation of FMT, the CCECAI score significantly increased in both groups compared to baseline (on tylosin treatment).

Dogs in the FMT group showed similar CCECAI scores during FMT treatment to the baseline (pre-tylosin), highlighting a possible therapeutic effect of FMT capsules, likely by modulation of the gut microbiota. One could argue that the therapeutic effect of tylosin can last after discontinuation, but the CCECAI scores of the placebo group increased after discontinuation of tylosin. A significant increase in CCECAI after discontinuation of FMT/placebo treatment compared to baseline might indicate transient engraftment and short therapeutic effects of FMT capsules, and the necessity of continuation of oral FMT treatment for a longer period.

Based on the fecal consistency score as our second efficacy variable, 83.3% (5/6) of dogs that received FMT for 4 weeks did not relapse compared to 50% (3/6) in the placebo group. However, this difference was not statistically significant. Numerically, there were trends for dogs receiving the placebo to have increases in their FCS in treatment visits compared to baseline (endoscopy visit), whereas dogs receiving FMT did not, suggesting that the fecal consistency was significantly more solid after receiving FMT compared to the placebo. Other studies also reported improved clinical scoring indices and fecal consistency in dogs with IBD after FMT [10,35], as well as in a dog with *Clostridium difficile*-associated diarrhea [36]. Since fecal consistency scoring was performed subjectively by the owners’/investigator’s opinion, fecal dry matter percentage was also examined to obtain more objective results of fecal consistency. We found a strong negative correlation between the FCS and FDM scores, which shows that the FDM percentage decreases when the fecal consistency score increases. If the visual interpretation of fecal consistency is considered as accurate as the fecal dry matter percentage, one might expect an even stronger correlation. A possible reason for the lack of an even stronger negative correlation could be that the total amount of defecated feces was not analyzed, but only a small portion (1 g) of each collected non-homogenous fecal sample. Therefore, there is a possibility of sampling fewer water-containing parts of feces.

In our trial, receiving probiotics, prebiotics, antibiotics, glucocorticoids, NSAIDs, and raw diet feeding were not permitted, and if a dog was on one of the prohibited items, the dog needed to stop taking it and wait for at least 4 weeks before the screening visit. However, one dog received a Canius supplement (containing Lactobacillus) for 1 week after stopping tylosin, and then the supplement was immediately stopped, and 3 weeks later, FMT treatment was started. Additionally, another dog in the placebo group received sour milk (containing *Lactobacillus acidophilus* and *Bifidobacterium*) for 1 week, and the owner did not realize this was not allowed during the trial. Six days later, the dog relapsed during placebo treatment. Therefore, it was released from the study. All other dogs continued to receive the same diet as before during the trial.

### 4.2. Impact of FMT on Microbiome Composition

We found that after 4 weeks of FMT treatment, the fecal microbiomes of TRE dogs mainly contained *Clostridium perfringens*, *Blautia hansenii*, *Escherichia coli*, *Faecalimonas umbilicata*, *Collinsella intestinalis*, *Bacteroides* sp., and *Romboutsia ilealis*. The presence of *C. perfringens* and *Escherichia coli* in the fecal microbiomes of TRE dogs should not cause alarm, as not all strains produce toxins or virulence factors [37] and are common residents of the mammalian gut microbiome. Genomic analysis of an *F. umbilicata* strain isolated from human feces revealed genes for acetate and vitamin B12 synthesis [38], which could be beneficial for the canine gut. Similarly, *Bacteroides* spp. are key fermenters of dietary carbohydrates into succinate, acetate, and propionate, which can serve as energy sources for the host [39].

Interestingly, the relative abundance of *Clostridium* and *Collinsella* increased in cats receiving oral capsule FMTs for their chronic vomiting [40]. Furthermore, dogs infected with Parvovirus have relatively more *Escherichia*, *Lachnoclostridium*, and *Ruminococcus* gnavus but fewer *Collinsella* than healthy dogs of comparable breeds and ages [41].

Differential abundance analysis indicated that, compared to baseline microbiomes pre-FMT, the fecal microbiomes of TRE dogs after 4 weeks of treatment showed statistically significant increases in the relative abundances of three bacterial taxa: *Clostridium dakarense*, *Clostridium paraputrificum*, and *Butyricicoccus pullicaecorum*. Only one of the three aforementioned taxa (*Butyricicoccus*) also increased in the fecal microbiomes of dogs that received a 25-day course of oral capsule FMTs for their chronic diarrhea, vomiting, or constipation [27]. This study also found that the relative abundances of *Faecalibacterium*, *Fusobacterium*, *Megamonas*, and *Sutterella* increased after FMT. Interestingly, *C*. *paraputrificum* was isolated and identified via MALDI-TOF from the feces of domestic dogs with lower gastrointestinal tract disorders in Brazil [42], but their relevance to canine health needs to be investigated more in-depth.

### 4.3. Impact of FMT on Microbiome Alpha and Beta Diversity

Following FMT, there was a trend towards increased alpha-diversity in TRE dogs at the treatment visit compared to the screening visit, although this increase was not statistically significant. This trend suggests that FMT may help in partially restoring microbial diversity in TRE dogs. The significant increase in alpha diversity from the screening and endoscopy visits to the post-treatment visit further supports the potential benefits of FMT in enhancing microbial diversity over time. Similar to that observed by Berlanda et al. (2021), we observed an improvement in the alpha diversity of dogs receiving FMT treatment [43]. Moreover, this improvement lasted until the post-treatment visit, confirming the results of the above-mentioned study. Although we did not see a significant difference in the dogs receiving FMT at the treatment visit compared to the other visits, this is in agreement with what has been observed by other researchers. For instance, Innocente et al. (2022) found no significant difference in pre- and post-FMT compared with any of the alpha diversity indices they considered [15]. Nevertheless, individual receivers generally showed a shift from pre-FMT microbiota. These changes differed according to the type of treatment response. This aligns with the variability in individual responses to FMT treatment observed in patients. However, it is noteworthy that similar increases in alpha diversity were observed in the placebo group, indicating that factors other than FMT, such as stopping tylosin treatment or natural progression, might have contributed to the observed changes. In a study by Manchester et al. (2019), tylosin treatment for 7 days in healthy dogs decreased fecal bacterial alpha diversity, which is characterized by decreases in the anaerobes *Fusobacteriaceae* and *Veillonellaceae* [44]. Fecal microbiota analysis at 14 and 56 days after stopping tylosin treatment showed partial recovery of decreased bacterial abundance. Another study of healthy dogs treated with tylosin for 7 days showed an altered abundance of most evaluated bacteria and a significant decrease in secondary bile acid concentrations on Day 7 in all dogs [45]. However, most parameters returned to baseline by Day 14 in all the dogs. In our study, the high alpha diversity after receiving FMT/placebo could be partially due to the natural recovery of microbiota after stopping tylosin treatment. However, the diversity increased more in the FMT group than in the placebo group, indicating that FMT may help partially restore microbial diversity in TRE dogs.

The analysis of beta diversity, using the Bray–Curtis dissimilarity index, provided additional insights into the microbial community structure among the different cohorts. PERMANOVA analysis revealed a significant overall difference in the microbial communities among the groups. TRE dogs receiving FMT showed distinct microbial communities compared with healthy dogs at all visit points, except for the post-treatment visit. This suggests that while FMT may help align the microbial community of TRE dogs closer to that of healthy dogs, complete normalization may not be achieved within the timeframe of the study. Interestingly, the placebo group also exhibited significant differences in beta diversity compared to healthy dogs, except at the post-treatment visit, where a trend towards significance was observed. This implies that placebo effects or cessation of tylosin treatment might have influenced the microbial composition in dogs with TRE.

A study investigating the microbiome responses to oral FMT capsules in a cohort of dogs with chronic digestive issues did not find an increase in alpha diversity of their gut microbiome after FMT [27], which is partially in contrast with the findings of our study since we saw a difference and a trend, and it also contrasts with previous studies’ findings conducted in dogs that took FMTs for their chronic enteropathy [36,43]. In another double-blinded, randomized clinical trial, 13 client-owned dogs with IBD received a one-time FMT/placebo via enema, in addition to their standard treatment (corticosteroid and hypoallergenic diet), and their fecal microbiome was examined 1 and 4 weeks after enema. Their results showed that the addition of FMT to standard therapy of IBD did not significantly change the fecal microbial diversity in recipients [14].

### 4.4. Efficacy of FMT in Regards to Bacterial Engraftment

We found that 30.4% of the bacterial strains (e.g., amplicon sequence variants) were engrafted in FMT recipients (engraftment rate range: 7.24–47.61%). This rate is significantly higher than that reported for a cohort of dogs receiving oral capsule FMTs for chronic diarrhea, vomiting, or constipation [27]. This is perhaps due to the fact that in the present study, dogs received tylosin before FMT treatment, which could have provided a “clean slate” and facilitated bacterial engraftment. Furthermore, in our trial, only dogs confirmed to have tylosin-responsive enteropathy were able to participate, whereas in the previously published study, the dogs may not have had a formal diagnosis.

The most commonly engrafted bacteria in FMT recipients were *Blautia hansenii*, *B. caecimuris*, *B. marasmi*, *Ruminococcus gnavus*, unclassified *Lachnoclostridium*, *Collinsella intestinalis*, and *Fournierella massiliensis*. These findings are consistent with and build upon those reported by Rojas et al. (2024), which found that many of the donor bacteria that were shared with FMT recipients were classified as uncultured *Lachnospiraceaea*, *Lachnoclostridium*, *Blautia*, *Ruminococcus torques*, *Fusobacterium*, and *Bacteroides* [27]. In the present study, full-length 16S rRNA gene sequencing was employed, which yielded species-level insights regarding bacterial engraftment.

Interestingly, *B. hansenii* appears to increase in the fecal microbiomes of elderly dogs receiving a daily probiotic feed additive [46]. *Collinsella* sp. appears to be a common resident of the gut microbiome in Border Collies and Labrador Retrievers [47]. Not much is known regarding *F. massiliensis*, other than they may have a beneficial role in the gut of its mammalian host via the production of butyrate, a short-chain fatty acid [48].

### 4.5. Adverse Effects and Study Implications

A recent Cochrane review of 12 studies on FMT in human inflammatory bowel disease reported moderate certainty evidence for AEs during FMT and very low certainty evidence for serious AEs [49]. In veterinary medicine, no FMT-related side effects have been observed in dogs with CE or IBD [14,35], and some short-lasting, self-limiting, and mild diarrhea or other GI signs have been observed in other FMT studies on dogs with CE [12,43]. In our study, a causal relationship between FMT and mild adverse events (AE) in two dogs during FMT treatment and unexpected severe AEs in two other dogs that occurred more than ten days after discontinuing FMT seems rather unlikely. FMT capsules for our study were made from the stools of one proven healthy dog donor. The collected feces from the donor dog underwent a strict screening for pathogens (e.g., toxin-producing *Clostridiodes difficile* or *Clostridium perfringens*, *Campylobacter* sp., *Escherichia coli*) to ensure that the FMT product is safe. Additionally, we cultured collected fecal samples from different time points of the dog with fibro-necrotic cholangitis and also from the remaining FMT capsules since none of the fecal samples or FMT capsules showed positive results for pathogenic or ESBL bacteria, a causal relationship between FMT and bacterial fibro-necrotic cholangitis seems unlikely.

By simplifying delivery and reducing the required manpower and infrastructure, oral administration is likely to make FMT an even more cost-effective treatment strategy. In addition, in a study by Kao et al. (2017), oral capsules were shown to be non-inferior to delivery by colonoscopy and represent an effective approach for the treatment of recurrent CDI in humans [50]. Similar to our clinical trial, some studies have successfully applied capsules containing freeze-dried feces to treat dogs with diarrhea or vomiting [27,51,52]. However, fecal microbiota can sometimes fail to reach their intestinal target after oral administration of FMT [50].

Despite the positive outcomes of FMT, the long-term effects of FMT are not sufficiently studied because of the non-standardized nature of the fecal matrix and the huge variability of microbiota between individual healthy subjects that serve as donors. Therefore, developing strategies to simulate FMT using characterized multi-species gut colonizers with identified phylogenetic diversity may be a promising alternative to FMT and mono- or multi-strain probiotics. Petrof et al. (2013) reported in humans the successful treatment of recurrent CDI using a FMT substitute constituted by 33 different purified intestinal bacteria isolated from one healthy donor [53]. Since the manipulation of intestinal microbiota has been a promising concept to prevent and treat different intestinal diseases, the pharmaceutical and food industries are interested in developing characterized communities of selected fecal bacteria, allowing a standardized, commercially prepared, stable, and reproducible FMT product.

### 4.6. Limitations

The present study had some limitations. The sample size was small, which limited the power of the analysis, and, therefore, the safety and efficacy conclusions that can be drawn. Since only 14 dogs were included in the 2.5-year period in the present trial, multicenter trials are encouraged to help recruit a larger sample size for future larger-scale studies of FMT efficacy and safety. Another reason for recruiting a small number of TRE dogs was the coronavirus pandemic restrictions during the trial. Leaving out the determination of basic serum cortisol and abdominal ultrasound from the diagnostic protocol might be considered a limitation since this could have led to missing dogs with GI signs due to hypoadrenocorticism or intestinal masses. However, it needs to be considered that dogs affected with such disorders are rather unlikely to respond to tylosin treatment alone, and with this were also improbable to enter the trial dog groups. While the exact optimal dosage of FMT for dogs has not been universally established, our approach was informed by existing research and aimed to ensure each dog received an appropriate and effective dose of donor fecal material [27,43,45]. However, this preliminary study helped to evaluate the feasibility and methodology of using freeze-dried oral FMT capsules in dogs with TRE, and it will shed light on future clinical trials with larger sample sizes to make more robust conclusions regarding the long-term efficacy and safety of FMT in canine patients with different CE subtypes.

## 5. Conclusions

In conclusion, our results showed that the efficacy of FMT was not significantly different than the placebo, which might be due to underpowering. After FMT treatment, there was a trend towards increased alpha diversity in TRE dogs at the treatment visit compared to the screening visit, which suggests that FMT may help in partially restoring microbial diversity in TRE dogs, although this increase did not reach statistical significance. In the post-treatment visit, fecal microbiome alpha diversity significantly increased compared to the screening and endoscopy visits, which further supports the potential benefits of FMT in enhancing microbial diversity over time. Furthermore, comparisons to the stool donor showed that, on average, 30.4% of donor strains were engrafted in FMT recipients. Our results suggest that oral FMT might be useful in restoring microbial diversity in dogs with TRE. However, further randomized controlled trials with larger sample sizes are needed to reliably determine the efficacy of oral FMT in dogs with TRE or CE.

## Figures and Tables

**Table 1:** Demographic data of dogs in the FMT or placebo groups.

		FMT (N = 7)	Placebo (N = 7)	Total (N = 14)	*p* Values
Variable		n (%)	n (%)	n (%)	
Age [years]	N	7	7	14	0.452
	Min	1	2	1	
	Median	2.0	4.0	2.5	
	Max	13	13	13	
Sex	Female	4 (57.1)	2 (28.6)	6 (42.9)	0.28
	Male	3 (42.9)	5 (71.4)	8 (57.1)	
Weight [kg]	N	7	7	14	0.729
	Min	6.4	9.8	6.4	
	Median	16.80	20.40	18.90	
	Max	49.4	34.7	49.4	
Neutered	No	3 (42.9)	4 (57.1)	7 (50.0)	0.592
	Yes	4 (57.1)	3 (42.9)	7 (50.0)	
Breed	Collie	1	3	4	
	Cavalier King Charles Spaniel	2	0	2	
	Australian Kelpie	1	0	1	
	Coton de tulear	0	1	1	
	Giant Poodle	1	0	1	
	Golden Retriever	1	0	1	
	Lagotto Romagnolo	0	1	1	
	Mixed breed	1	0	1	
	Schapendoes	0	1	1	
	White Shepherd	0	1	1	

**Table 2:** Median (range) of fecal dry matter (FDM) percentage in TRE dogs of both FMT and placebo groups.

Group		Visit						
		Screening	Inclusion	Endoscopy	Treatment		Post-Treatment	
					Relapse	No Relapse	Relapse	No Relapse
FMT	Number of dogs	7	7	7	2	5	1	2
	Median (range) of FDM percentage	36.75 (28.3–49.6)	30.45 (22.6–34.6)	37.85 (32.1–49)	34.73 (27.95–41.5)	36.95 (29–38.3)	38	37.98 (36.2–39.75)
Placebo	Number of dogs	6	6	6	3	3	0	3
	Median (range) of FDM percentage	36.6 (28.9–45)	28.60 (21.9–37.5)	38.58 (33.7–41.2)	27.65 (25.55–28.75)	35.05 (29.25–36.25)		40.95 (36.25–42.95)

## Data Availability

The original contributions presented in the study are included in the article/supplementary material, further inquiries can be directed to the corresponding author.

## Supplementary Materials

The following supporting information can be downloaded at: https://www.mdpi.com/article/10.3390/vetsci11090439/s1, Figure S1: Flow chart of the screening phase to verify the diagnosis of tylosin responsive enteropathy (TRE) in dogs, which was required for participation in the clinical trial; Figure S2: Results of allocation and response to FMT/placebo treatment of TRE dogs; Figure S3: Fecal microbiome composition (Genus-level) of dogs receiving FMT or Placebo treatment. Samples are faceted by individual and ordered by time point (1—screening, 2—inclusion, 3—endoscopy, 4—treatment, 5—post-treatment). Not all dogs have all 5 samples. The relative abundances of bacterial genera with mean relative abundances >0.6% are shown, while all others are collapsed into the “Other” category; Figure S4: Fecal microbiome composition (Species-level) of dogs receiving FMT or placebo treatment. Samples are faceted by individual and ordered by time point (1—screening, 2—inclusion, 3—endoscopy, 4—treatment, 5—post-treatment). Not all dogs have all 5 samples. The relative abundances of bacterial genera with mean relative abundances > 1.15% are shown, while all others are collapsed into the “Other” category; Table S1: Inclusion and exclusion criteria; Table S2: Demographic data of healthy dogs whose microbiomes served as a reference for comparison with TRE dogs; Table S3: Average relative abundances of bacterial genera for dogs in the FMT or placebo group, and for donors and healthy dogs. Screening, inclusion, and endoscopy samples were categorized as “pre” while treatment and post-treatment samples were categorized as “post.”; Table S4: Average relative abundances of bacterial species for dogs in the FMT or placebo group, and for donors and healthy dogs. Screening, inclusion, and endoscopy samples were categorized as “pre” while treatment and post-treatment samples were categorized as “post.”; Table S5: Pairwise comparison of Shannon Index between all time points in the FMT dogs and Healthy dogs; Table S6: Pairwise comparison of Shannon Index between all time points in the Placebo dogs and Healthy dogs; Table S7: Pairwise comparison of PERMANOVA analysis of Bray-Curtis dissimilarity index between all time points in the FMT dogs and Healthy dogs; Table S8: Pairwise comparison of PERMANOVA analysis of Bray-Curtis dissimilarity index between all time points in the Placebo dogs and Healthy dogs; Table S9: Engraftment of Donor bacteria after FMT. ASV engraftment rates were calculated by dividing the number of ASVs shared between FMT recipients postFMT and their stool donors (excluding taxa shared between preFMT samples and donors) by the total number of ASVs in the donor sample (excluding any taxa shared with preFMT samples). 100% Engraftment would indicate that all of the donor ASVs with the capacity to engraft did indeed engraft; Table S10: Severity score of total histopathologic changes in the intestinal segments of TRE dogs in FMT and placebo groups; Table S11: The diet information of each dog with TRE participated in the FMT/placebo trial; Table S12: The individual data of the two FDM% measurements, their mean values, standard deviations, and coefficient of variation (CV%). FDM%: fecal dry matter percentage; SD: standard deviation.
