# Supplementary material for: Oral Fecal Microbiota Transplantation in Dogs with Tylosin-Responsive Enteropathy—A Proof-of-Concept Study
Source: Vet Sci. 2024 Sep 18;11(9):439. doi: 10.3390/vetsci11090439 (PMC11435887; doi:10.3390/vetsci11090439)
Supplement: Supplementary file 1 [file vetsci-11-00439-s001.zip › Supplementary Figures-Mohsen Hanifeh.pdf]

Supplementary Figures for:

Oral fecal microbiota transplantation in dogs with tylosin responsive enteropathy- A proof-of-concept study

Mohsen Hanifeh[1,*], Elisa Scarsella[2], Connie A. Rojas[2], Mirja Huhtinen[3], Tarmo Laine[3], Holly H. Ganz[2], Thomas Spillmann[1]

Correspondence: mohsen.hanifeh@helsinki.fi

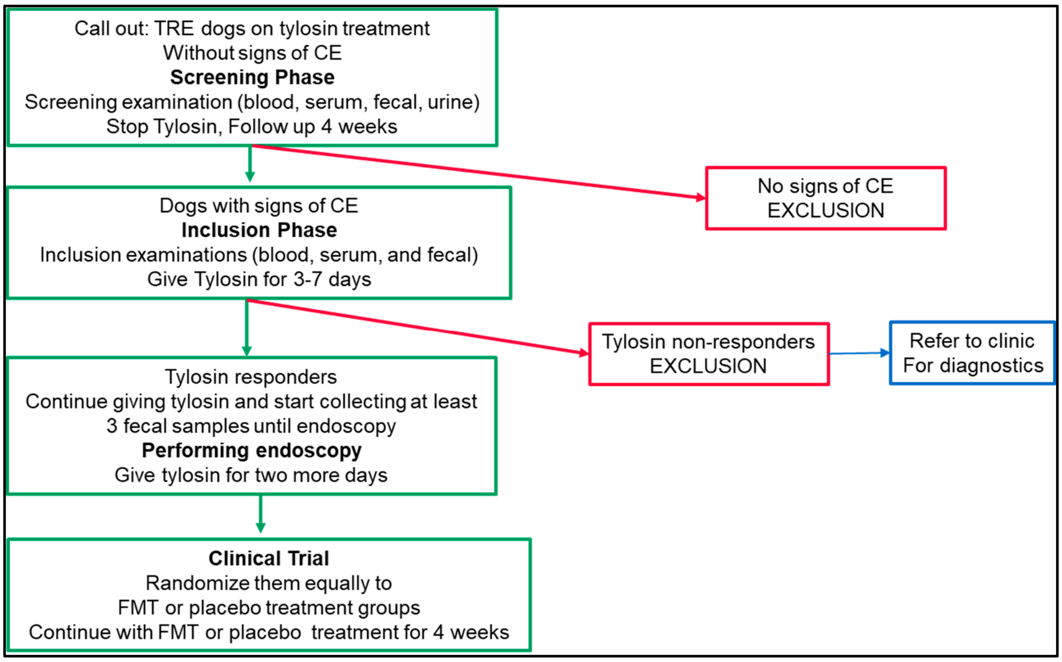

Figure S1. Flow chart of the screening phase to verify the diagnosis of tylosin responsive enteropathy (TRE) in dogs, which was required for participation in the clinical trial.

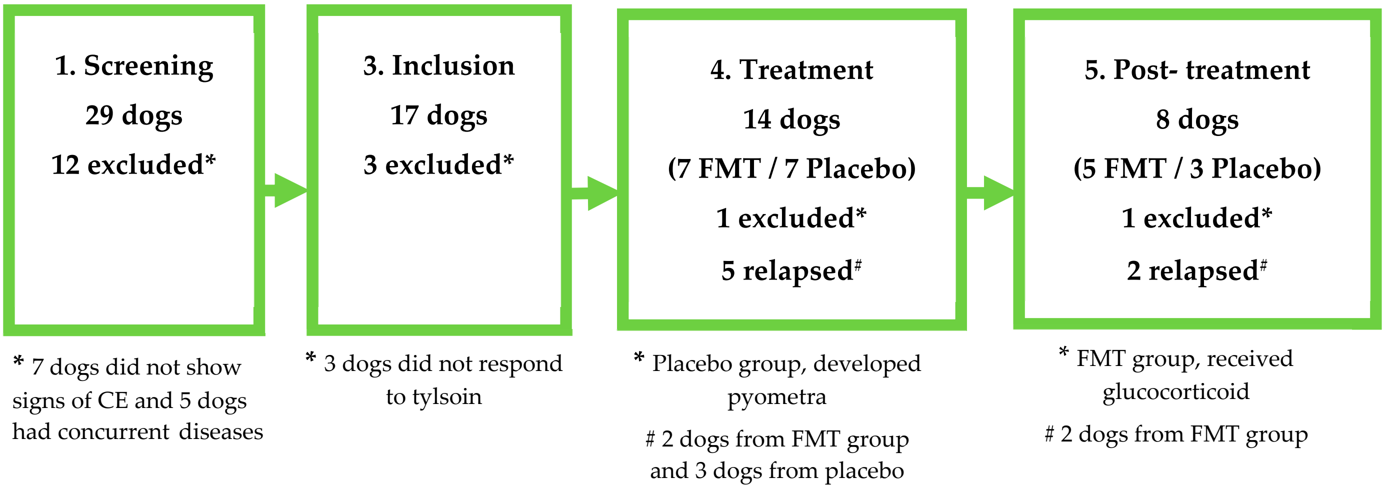

Figure S2. Results of allocation and response to FMT/placebo treatment of TRE dogs.

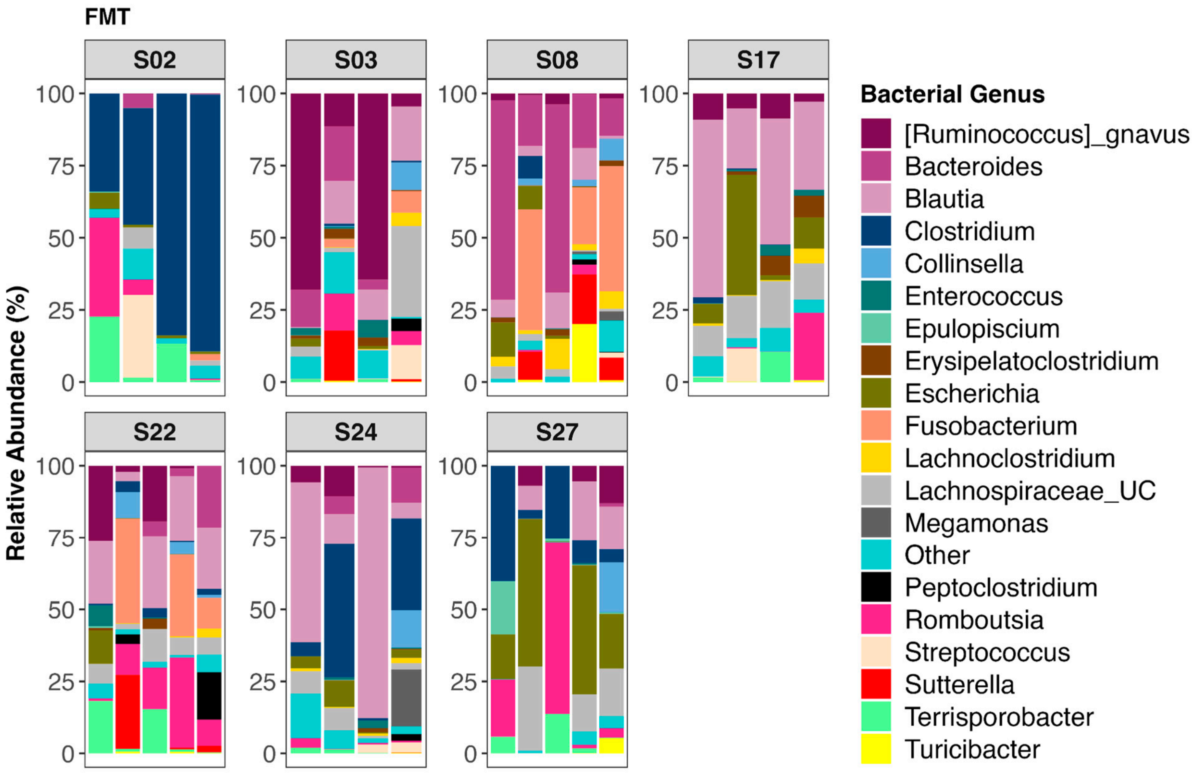

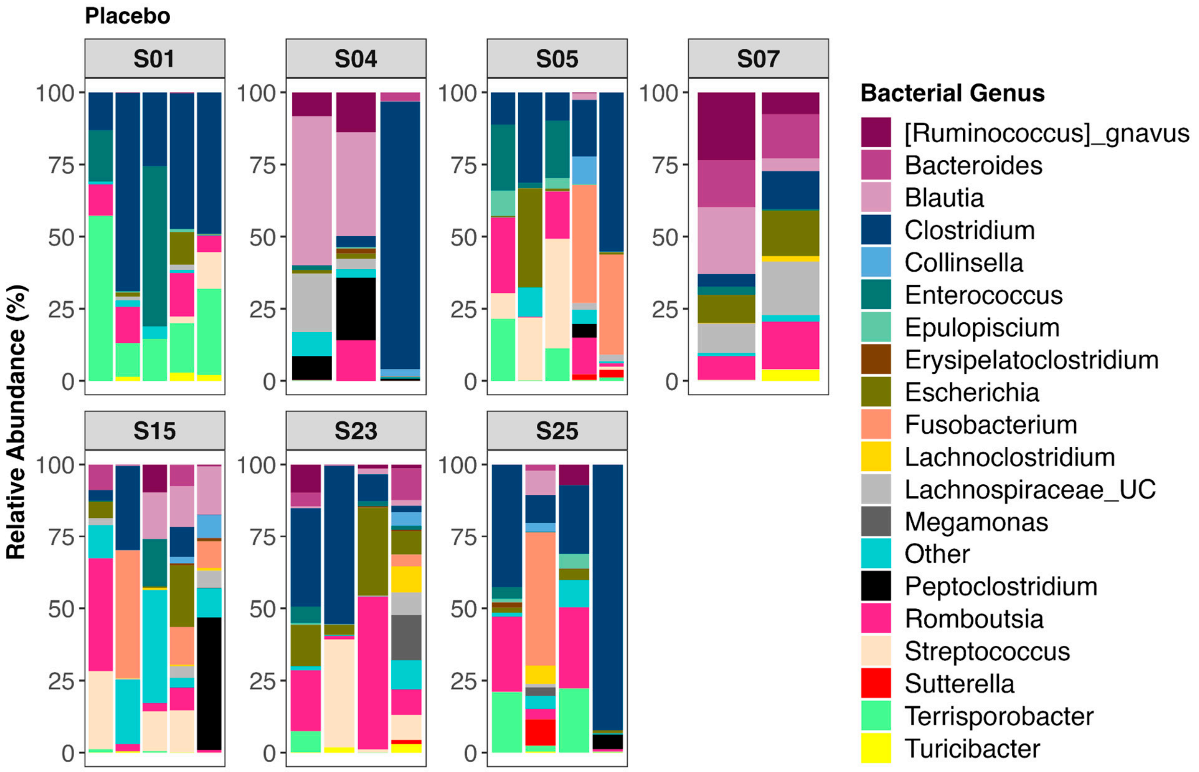

Figure S3. Fecal microbiome composition (Genus-level) of dogs receiving FMT or Placebo treatment. Samples are faceted by individual and ordered by time point (1 - screening, 2 - inclusion, 3- endoscopy, 4- treatment, 5 – post-treatment). Not all dogs have all 5 samples. The relative abundances of bacterial genera with mean relative abundances >0.6% are shown, while all others are collapsed into the “Other” category.

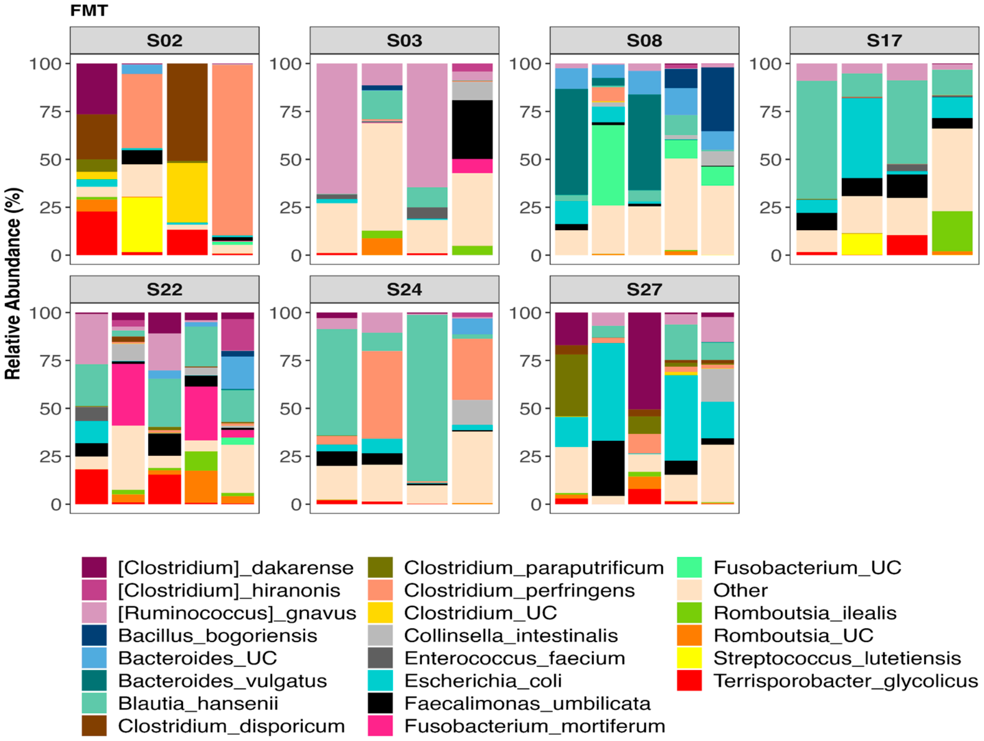
FMT
S02
S03
S08
S17
S22
S24
S27
Relative Abundance (%)
0
25
50
75
100
[Clostridium]_dakarense
[Clostridium]_hiranonis
[Ruminococcus]_gnavus
Bacillus_bogoriensis
Bacteroides_UC
Bacteroides_vulgatus
Blautia_hansenii
Clostridium_disporicum
Clostridium_paraputrificum
Clostridium_perfringens
Clostridium_UC
Collinsella_intestinalis
Enterococcus_faecium
Escherichia_coli
Faecalimonas_umbilicata
Fusobacterium_mortiferum
Fusobacterium_UC
Other
Romboutsia_ilealis
Romboutsia_UC
Streptococcus_lutetiensis
Terrisporobacter_glycolicus

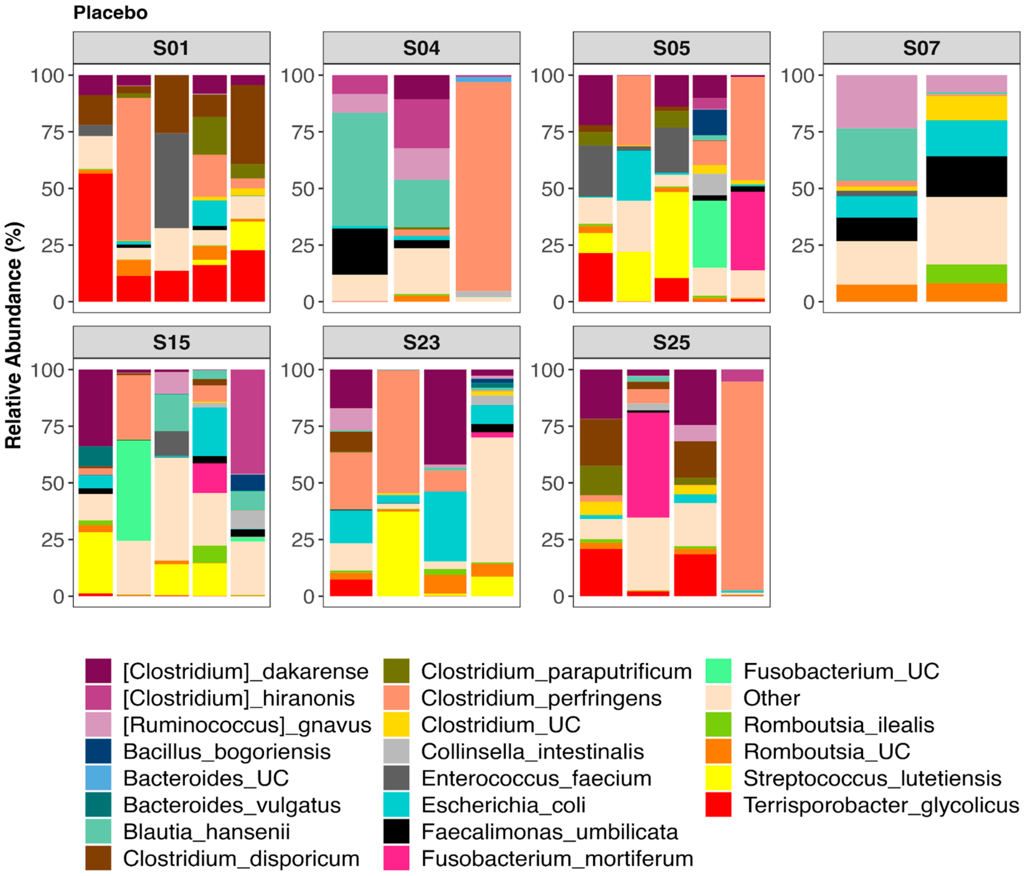
Placebo
S01
S04
S05
S07
S15
S23
S25
Relative Abundance (%)
0
25
50
75
100
[Clostridium]_dakarense
[Clostridium]_hiranonis
[Ruminococcus]_gnavus
Bacillus_bogoriensis
Bacteroides_UC
Bacteroides_vulgatus
Blautia_hansenii
Clostridium_disporicum
Clostridium_paraputrificum
Clostridium_perfringens
Clostridium_UC
Collinsella_intestinalis
Enterococcus_faecium
Escherichia_coli
Faecalimonas_umbilicata
Fusobacterium_mortiferum
Fusobacterium_UC
Other
Romboutsia_ilealis
Romboutsia_UC
Streptococcus_lutetiensis
Terrisporobacter_glycolicus

Figure S4. Fecal microbiome composition (Species-level) of dogs receiving FMT or placebo treatment. Samples are faceted by individual and ordered by time point (1 - screening, 2 - inclusion, 3- endoscopy, 4- treatment, 5 – post-treatment). Not all dogs have all 5 samples. The relative abundances of bacterial genera with mean relative abundances >1.15% are shown, while all others are collapsed into the "Other" category.
