# Supplementary material for: Oral Fecal Microbiota Transplantation in Dogs with Tylosin-Responsive Enteropathy—A Proof-of-Concept Study
Source: Vet Sci. 2024 Sep 18;11(9):439. doi: 10.3390/vetsci11090439 (PMC11435887; doi:10.3390/vetsci11090439)
Supplement: Supplementary file 1 [file vetsci-11-00439-s001.zip › Supplementary Tables-Mohsen Hanifeh.pdf]

Supplementary Tables for:

Oral fecal microbiota transplantation in dogs with tylosin responsive enteropathy- A proof-of-concept study

Mohsen Hanifeh[1,*], Elisa Scarsella[2], Connie A. Rojas[2], Mirja Huhtinen[3], Tarmo Laine[3], Holly H. Ganz[2], Thomas Spillmann[1]

Correspondence: mohsen.hanifeh@helsinki.fi

Table S1. Inclusion and exclusion criteria

Inclusion criteria	Exclusion criteria
• Age over one year old.<br>• Body weight > 5 kg<br>• Dog has not received systemic corticosteroids, non-steroidal anti-inflammatory drugs or any other antibiotics but tylosin within 30 days preceding or during the trial.<br>• Dog has been previously treated successfully with tylosin because of signs of chronic enteropathy (CE) such as chronic vomiting, chronic diarrhea, decreased appetite and weight loss lasting more than 3 weeks.<br>• Dog was on tylosin medication and had no clinical signs of CE at the beginning of the trial.	• Pregnant or lactating dog.<br>• Evidence of significant systematic or organ related diseases, such as renal diseases, diabetes mellitus, exocrine pancreatic insufficiency, neoplasia, intestinal obstructive disease, neuropathies, heart diseases, etc.<br>• Dogs with TRE that did not respond to tylosin treatment after 3-7 days and dogs that did not experience a relapse after discontinuing the tylosin treatment within 4 weeks of follow up.<br>• Dog is eating raw food diets (Bones and *Raw Food* = BARF*) (49)*<br>• Concurrent participation in another clinical study<br>• Any other reasons that can interfere with the conduct of the study (e.g., owner compliance)

Table S2. Demographic data of healthy dogs whose microbiomes served as a reference for comparison with TRE dogs.

**Dogs**		**Total (N=xx)**
**Variable**		**n (%)**
**Age [years]**	N	30 (100%)
	Min	0.9
	Median	4.05
	Max	12.2
**Sex**	Female	12 (40%)
	Male	18 (60%)
**Weight [kg]**	N	30 (100%)
	Min	6
	Median	15.5
	Max	55
**Neutered**	No	13 (43%)
	Yes	17 (57%)
**Breed**	Border Collie	3
	Dachshund	3
	Siberian Husky	3
	Border Terrier	2
	Golden Retriever	2
	Jack Russell Terrier	2
	Lagotto Romagnolo	2
	Miniature Poodle	2
	Nova Scotia Duck Tolling Retriever	2
	American Pit Bull Terrier	1
	Cane Corso	1
	Finnish Lapphund	1
	German Wirehaired Pointer	1
	Labrador Retriever	1
	Lancashire Heeler	1
	Parson Russell Terrier	1
	Pug	1
	Shetland Sheepdog	1

Table S3. Average relative abundances of bacterial genera for dogs in the FMT or placebo group, and for donors and healthy dogs. Screening, inclusion, and endoscopy samples were categorized as "pre" while treatment and post-treatment samples were categorized as "post."

**Bacterial Genus**	**Donor average**	**Healthy average**	**FMT-pre average**	**FMT-post average**	**Placebo-pre average**	**Placebo-post average**
Blautia	2.6371	9.9144	18.3403	14.6241	7.527	3.9246
Clostridium	0.2656	5.4131	14.2036	13.6827	20.4988	40.9567
Fusobacterium	0.1177	14.9381	3.8694	11.1828	4.773	11.3365
Lachnospiraceae_UC	4.2628	6.9992	6.1803	9.1248	3.099	2.7382
Escherichia	0.021	0.7628	8.3615	7.8741	6.6419	4.7686
Romboutsia	0.021	7.3026	7.7835	7.7634	14.8778	5.9226
Bacteroides	1.2947	3.7037	9.6958	6.9098	2.517	2.4175
Collinsella	0.3371	2.785	0.5548	5.4866	0.172	3.0148
[Ruminococcus] gnavus	0.8243	1.0815	11.6832	2.9755	4.289	0.2866
Sutterella	0.1008	1.5471	2.5083	2.8673	0.4787	0.6704
Turicibacter	0.0042	0.4855	0.0902	2.7724	0.4176	0.9066
Peptoclostridium	1.5015	8.3594	0.1638	2.5291	1.5758	6.2799
Megamonas	30.1567	8.6326	0.0031	2.3959	0.1602	1.8007
Lachnoclostridium	0.8155	0.9334	0.9129	2.3163	0.4814	1.1531
Streptococcus	15.0621	8.703	2.0818	1.7025	7.8149	4.3547
Erysipelatoclostridiu m	0.0605	0.3271	1.2241	1.0555	0.2426	0.264
Faecalibacterium	0.1293	0.0085	0	0.8326	0	0.0076
Faecalitalea	0.1523	0.0157	0.9243	0.4873	0.0463	0.0269
Fournierella	0.0907	0.2628	0	0.4715	0.0155	0.26
Campylobacter	0	0.0151	0.0151	0.3991	0	0.0419
Terrisporobacter	0	0.1214	5.2086	0.3753	9.013	5.4232
Tyzzerella	0.0706	0.3763	0.4869	0.3722	0.7698	0.0848
Enterococcus	0	0.3925	1.1934	0.3036	7.9553	0.1739
[Ruminococcus]_torqu es	0	0.1765	0.0253	0.1668	0	0
[Eubacterium]_tenue	0	0.0304	0.1974	0.1468	0.4104	0.2959
Oscillospiraceae_UCG -002	0.396	0.0669	0	0.1273	0	0.0142
Clostridioides	0	0	0.4588	0.1079	0.7821	0.0027
Anaerostipes	0	0.051	0.364	0.1002	0.1584	0

Paeniclostridium	0	0.1419	0.3237	0.0914	0.2849	0.1434
Lachnospira	0	0.1976	0	0.0872	0	0.0247
Harryflintia	0	0.0023	0	0.067	0	0
Roseburia	0.0914	0.0042	0	0.0555	0.1109	0.0085
Butyricicoccus	0.2609	0.035	0.0326	0.0553	0.0356	0.0097
Epulopiscium	0	0.0152	0.9865	0.054	1.0525	0.1795
Hydrogenoanaerobact erium	0	0	0	0.039	0	0
Ralstonia	0.0547	0	0.1756	0.0379	0.0883	0.0139
Lachnospiraceae_UC G-004	0	0	0	0.0353	0	0.0209
Flavonifractor	0	0	0.1829	0.0272	0.0084	0
[Clostridium]_innocu um	0	0	0.4436	0.026	1.1234	0.0106
Arthromitus	0	0.0057	0	0.0259	0.0012	0
Bilophila	0	0	0.0034	0.0256	0.027	0
Parabacteroides	0	0	0.0185	0.0231	0	0.0133
Oscillospirales_UC	0.0762	0.007	0	0.022	0	0
Ruminococcaceae_ UC	0.1599	0.0016	0	0.0203	0.0003	0.1233
Oscillospiraceae_UC	0	0	0	0.0183	0	0
Tessaracoccus	0	0.0008	0.0057	0.018	0.0011	0.0012
Hungateiclostridiacea e_ UC	0.0457	0.0421	0	0.0165	0.0006	0.3529
Paraeggerthella	0	0	0	0.0146	0	0
Caballeronia	0	0.0706	0.0976	0.013	0.0305	0.0085
Brevundimonas	0	0	0.0335	0.0121	0.2407	0.0045
Gemella	0	0	0.0061	0.0074	0	0.001
Propionibacterium	0	0	0.0021	0.0064	0	0
Rhodococcus	0	0.0203	0.032	0.0056	0.0103	0.0023
Luteimonas	0	0	0	0.0055	0	0
Mycobacterium	0	0	0	0.0046	0	0
Corynebacterium	0	0.0026	0.1164	0.0045	0	0
Actinomyces	0	0.0033	0.0025	0.0045	0.0025	0.0029
Allobaculum	0.4125	1.506	0	0.0044	0	0
Oscillibacter	0	0	0	0.0043	0	0
Aerococcaceae_ UC	0	0	0.0112	0.0035	0.0006	0.0055

Holdemanella	0.3577	2.6903	0	0.0026	0.0021	0
Propionibacteriaceae_ UC	0	0	0	0.0025	0	0
Odoribacter	0	0	0	0.0018	0	0
Acinetobacter	0	0	0.039	0.0014	0.0315	0
Anaerostignum	0	0.0021	0.0324	0.0012	0	0.0016
Brachybacterium	0	0	0	0.0008	0	0
Euzebya	0	0	0.0052	0.0004	0	0
Prevotella_9	12.431	4.0023	0	0	0	0.503
Bacteroidales_UC	5.8183	0	0	0	0	0
Ruminococcus	3.5032	0	0	0	0	0
Treponema	3.3128	0	0	0	0	0
Bacteria_UC	1.843	0	0.021	0	0.0037	0
Prevotellaceae_ UC	1.6678	0	0	0	0	0
Oscillospiraceae_ UC	1.287	0	0	0	0	0
Fibrobacter	1.1652	0	0	0	0	0
Catenibacterium	0.7028	0.4095	0	0	0	0
Colidextribacter	0.6473	0	0	0	0	0
Prevotella_7	0.6397	0	0	0	0	0
Prevotella	0.594	0	0	0	0	0
Bacilli_UC	0.5559	0	0	0	0	0
Succiniclasticum	0.5255	0.0796	0	0	0	0
Acidaminococcaceae_ UC	0.468	0.1315	0	0	0	0
Christensenellaceae_R -7	0.4265	0	0.0014	0	0	0
[Eubacterium]_hallii	0.4112	0	0	0	0	0
Bacteroidia_UC	0.3199	0	0	0	0	0
Coprobacter	0.2589	0	0	0	0	0
Alloprevotella	0.2361	1.2075	0	0	0	0.2023
Ligilactobacillus	0.2056	0	0	0	0.0028	0
Rhizobiales_UC	0.2056	0	0	0	0	0
Anaerovoracaceae_ UC	0.1828	0	0	0	0	0
Sarcina	0.1752	3.8873	0	0	0.117	0.0319
Erysipelatoclostridiace	0.1752	0	0	0	0	0

ae_ UC						
Saccharofermentans	0.1675	0	0	0	0	0
Clostridia_UC	0.1599	0	0	0	0	0
Schwartzia	0.1371	0	0	0	0	0
Stoquefichus	0.1295	0.2023	0	0	0.0535	0.0228
Sporobacter	0.1218	0	0	0	0	0
Caproiciproducens	0.1142	0	0	0	0	0
Gastranaerophilales	0.1142	0	0	0	0	0
Oligosphaera	0.1066	0	0	0	0	0
Akkermansia	0.099	0	0	0	0	0
Kiritimatiella	0.099	0	0	0	0	0
Eggerthellaceae_ UC	0.0914	0	0.005	0	0	0
Pseudobutyrivibrio	0.0914	0	0	0	0	0
Desulfonispora	0.0838	0	0	0	0	0
Catabacter	0.0762	0	0	0	0	0
Others	--------	--------	--------	--------	--------	--------

Table S4. Average relative abundances of bacterial species for dogs in the FMT or placebo group, and for donors and healthy dogs. Screening, inclusion, and endoscopy samples were categorized as "pre" while treatment and post-treatment samples were categorized as "post."

**Bacterial species**	**Donor average**	**Healthy average**	**FMT-pre average**	**FMT-post average**	**Placebo-pre average**	**Placebo-post average**
Clostridium perfringens	0.0042	2.8409	5.3886	12.7148	12.026	30.0857
[Clostridium] hiranonis	1.5015	8.3594	0.1638	2.5291	1.5758	6.2799
Fusobacterium mortiferum	0.0227	0.3016	1.5479	3.9895	2.438	5.5883
Clostridium disporicum	0	0.5545	4.1142	0.4289	5.145	5.3352
Escherichia coli	0.021	0.4659	8.0903	7.8448	5.9709	4.744
Terrisporobacter glycolicus	0	0.1024	4.7993	0.3147	8.6377	4.5123
Streptococcus lutetiensis	11.1388	5.3722	1.8885	0.011	7.7782	4.2355

Fusobacterium UC	0.095	8.0327	1.9965	2.5298	2.3346	3.5047
Collinsella intestinalis	0.2	2.7713	0.5548	5.4866	0.172	2.9788
[Clostridium] dakarense	0	0.9088	5.4086	1.2031	10.7731	2.952
Clostridium paraputrificum	0	0.0072	2.4734	0.3256	1.7335	2.5707
Bacillus bogoriensis	0	6.6038	0.1214	4.6316	0.0004	2.2436
Faecalimonas umbilicata	0.1522	6.4913	5.0992	5.6536	3.0398	1.87
Megamonas funiformis	30.1567	8.6326	0.0031	2.3959	0.1602	1.8007
Blautia hansenii	1.2228	2.8313	17.1878	9.2359	6.0806	1.7555
Romboutsia UC	0.0084	5.3178	1.4913	2.5957	2.894	1.7209
Clostridium UC	0.2285	0.2308	1.7316	0.2021	1.2919	1.4631
Clostridium colicanis	0	0.734	0.0104	0	0.0927	1.446
Romboutsia ilealis	0.0126	0.9729	0.6431	3.9197	1.1005	1.1502
Lachnoclostridium UC	0.8155	0.844	0.6687	1.9986	0.3606	1.0902
Bacteroides coprocola	0.0781	0.1695	0	0	0.0004	1.0802
Blautia UC	0.6552	4.3192	0.8926	1.2303	0.3551	1.0405
Terrisporobacter UC	0	0.0107	0.4093	0.0495	0.3752	0.9109
Turicibacter sanguinis	0.0042	0.4855	0.0902	2.7724	0.4176	0.9066
Lachnospiraceae UC	4.1106	0.508	1.0811	3.4712	0.0592	0.8681
Sutterella stercoricanis	0.1008	1.5471	1.2715	2.5798	0.4787	0.6704
Blautia marasmi	0.2125	0.5485	0.2469	1.434	0.9947	0.6654
Bacteroides plebeius	0.393	0.2972	0.4496	1.3796	0.0076	0.6339

Prevotella copri	12.431	4.0023	0	0	0	0.503
[Eubacterium] tenue	0	0.0435	0.2058	0.1671	0.513	0.3777
Hungateiclostridiaceae UC	0.0457	0.0421	0	0.0165	0.0006	0.3529
Parasutterella secunda	0	0.0481	0	0	1.514	0.3149
[Ruminococcus] gnavus	0.8243	1.0815	11.6832	2.9755	4.289	0.2866
Bacteroides vulgatus	0	0.4804	5.2117	0.076	0.4849	0.2743
Bacteroides UC	0.7704	2.665	1.8887	5.1289	0.0012	0.2625
Blautia glucerasea	0.0151	1.8032	0.01	0.5365	0.0302	0.2617
Fournierella massiliensis	0.0907	0.2628	0	0.4715	0.0155	0.26
Phascolarctobacterium faecium	0	0	0.0114	0	0	0.2466
Alloprevotella rava	0.2361	1.2075	0	0	0	0.2023
Blautia caecimuris	0.1017	0.3128	0.0029	2.1874	0.0664	0.2015
Niameybacter massiliensis	0	0.0152	0.9865	0.054	1.0525	0.1795
Enterococcus faecalis	0	0.0066	0.1735	0	2.1412	0.1727
[Clostridium] spiroforme	0.0605	0.2472	0.2088	0.7324	0.0006	0.1549
Rothia endophytica	0	0	0	0	0.0025	0.1383
Peptostreptococcaceae UC	0	0.7488	0	0	0.0347	0.1239
Ruminococcaceae UC	0.1599	0.0016	0	0.0203	0.0003	0.1233
Erysipelatoclostridium ramosum	0	0.0473	0.8106	0.3231	0.242	0.1053
Romboutsia hominis	0	0.1031	0.2405	0.0449	0.1101	0.0995

Streptococcus macedonicus	0	1.1424	0	0.1671	0	0.0994
Klebsiella pneumoniae	0	0	0.0688	0	0.1677	0.0878
Providencia alcalifaciens	0	0	0	0	0	0.086
Bacteroides caecigallinarum	0	0.0392	0	0	0	0.0816
Tyzzerella nexilis	0	0.0282	0.4495	0.1389	0.7252	0.0803
Metabacterium polyspora	0	0.0538	0.011	0	0	0.0781
[Clostridium] glycyrrhizinilyticum	0	0.0894	0.2442	0.3177	0.0351	0.0629
Paeniclostridium UC	0	0.0067	0.3153	0.0711	0.1357	0.0615
Bacteroides stercoris	0	0	0	0.1959	0.1022	0.0532
Cellulosilyticum UC	0	0.016	0	0	0	0.042
Clostridium saudiense	0	0.0935	0.0007	0.0096	0.0123	0.0326
Sarcina maxima	0.1752	2.7975	0	0	0	0.0319
Bacteroides thetaiotaomicron	0	0	0	0.0018	0.2577	0.0266
Campylobacter jejuni	0	0	0	0	0	0.0254
Lactobacillus rogosae	0	0.0063	0	0	0	0.0247
Stoquefichus	0.1295	0.2023	0	0	0.0535	0.0228
Allisonella histaminiformans	0	0	0	0	0	0.0213
[Eubacterium] hallii	0.4112	0	0	0.0353	0	0.0209
Clostridium tertium	0	0.0719	0.3748	0	0.1801	0.0203
Collinsella stercoris	0	0	0	0	0	0.0186
Escherichia UC	0	0.0942	0.2229	0.0277	0.671	0.0182

Absiella dolichum	0	0.008	0.7003	0.2267	0.0451	0.0169
Campylobacter upsaliensis	0	0.0151	0.0151	0.3991	0	0.0165
Collinsella tanakaei	0	0.0041	0	0	0	0.016
Staphylococcus pseudintermedius	0	0.0708	0.0049	0	0.0657	0.0146
Trichuris trichiura	0.396	0.0669	0	0.1273	0	0.0142
Ralstonia insidiosa	0.0547	0	0.1756	0.0379	0.0883	0.0139
Parabacteroides merdae	0	0	0.0185	0	0	0.0133
Canibacter oris	0	0	0.0194	0	0	0.0129
[Clostridium] innocuum	0	0	0.4436	0.026	1.1234	0.0106
Absiella argi	0	0.0077	0.224	0.2606	0.0012	0.0101
Butyricicoccus pullicaecorum	0.2609	0.0302	0.0326	0.0553	0.0356	0.0097
Streptococcus canis	0	0.0075	0.0046	0	0	0.0096
Caballeronia sordidicola	0	0.0706	0.0976	0.013	0.0305	0.0085
Roseburia UC	0.0533	0.0042	0	0.0458	0.1109	0.0085
Faecalibacterium prausnitzii	0.1293	0.0085	0	0.8326	0	0.0076
Lactobacillus buchneri	0	0	0.0084	0	0	0.0068
Kocuria atrinae	0	0	0	0	0	0.0066
Escherichia albertii	0	0	0	0	0	0.0056
Aerococcaceae UC	0	0	0.0112	0.0035	0.0006	0.0055
Bacteroides dorei	0	0	0.0447	0.0051	0.3958	0.0051
Brevundimonas vesicularis	0	0	0.0335	0.0121	0.2407	0.0045

Streptococcus minor	0	0.0254	0.0296	0	0.003	0.0039
[Clostridium] saccharogumia	0	0.0326	0	0	0	0.0038
Streptococcus salivarius	0	0.0117	0.1169	1.0033	0.0153	0.0031
Clostridium baratii	0	0.0184	0.1099	0.0017	0.0173	0.0031
[Clostridium] colinum	0.0706	0.3481	0.0374	0.2173	0.0257	0.003
Clostridioides difficile	0	0	0.4072	0.0968	0.6502	0.0027
Rhodococcus fascians	0	0.0203	0.032	0.0056	0.0103	0.0023
Streptococcus alactolyticus	0	0.4102	0	0.3282	0	0.002
Leuconostoc gelidum	0	0	0.0037	0	0	0.0018
Anaerotignum aminivorans	0	0.0021	0.0324	0.0012	0	0.0016
Rothia UC	0	0	0	0	0	0.0016
Anaerotignum lactatifermentans	0	0	0	0.0161	0	0.0015
Collinsella provencensis	0	0	0	0	0	0.0014
Rothia nasimurium	0	0	0	0	0	0.0014
Enterococcus faecium	0	0.0586	0.9923	0.1092	5.547	0.0012
Streptococcus COT 279	0	0	0.0085	0	0.0116	0.0012
Tessaracoccus UC	0	0.0008	0.0057	0.018	0.0011	0.0012
Buchananella hordeovulneris	0	0.0005	0	0.0012	0	0.0012
Gemella palaticanis	0	0	0.0061	0.0074	0	0.001
Actinomyces COT 374	0	0.0007	0	0	0.002	0.0009
Schaalia canis	0	0.0021	0.0022	0.0018	0.0003	0.0008

Escherichia marmotae	0	0.2027	0	0.0017	0	0.0008
Moranbacteria	0	0	0	0	0.0003	0.0004
Comamonadaceae UC	0	0	0	0	0	0.0004
Peptostreptococcus canis	0	0.0026	0	0	0.0008	0.0002
Bacteroides ovatus	0	0	1.9781	0	0.3899	0
Sutterella parvirubra	0	0	1.2227	0.064	0	0
Anaerostipes caccae	0	0	0.364	0.0912	0.1584	0
Anaerobiospirillum succiniciproducens	0	0	0.3262	0	0	0
Massiliomicrobiota timonensis	0	0	0.2046	0	0	0
Fusobacterium varium	0	0	0.2036	0.0319	0	0
Flavonifractor plautii	0	0	0.1829	0.0272	0.0084	0
Bacteroides xylanisolvens	0	0	0.112	0	0.0934	0
Lactococcus lactis	0	0.1416	0.1019	0	0	0
Helicobacter rappini	0	0.0045	0.1	0	0	0
Corynebacterium UC	0	0.0026	0.0857	0.0045	0	0
Clostridioides UC	0	0	0.0515	0.0111	0.1319	0
Leuconostoc lactis	0	0	0.0511	0	0	0
Escherichia ruysiae	0	0	0.0482	0	0	0
Acinetobacter johnsonii	0	0	0.039	0.0014	0.0315	0
Leuconostoc mesenteroides	0	0	0.035	0	0	0

Streptococcus UC	0	0.0691	0.0335	0.1929	0.0068	0
[Ruminococcus] torques UC	0	0.1765	0.0253	0.0645	0	0
Bacteria UC	1.843	0	0.021	0	0.0037	0
Methylorubrum rhodesianum	0	0	0.0195	0	0	0
Corynebacterium tuberculostearicum	0	0	0.0191	0	0	0
Enterococcus UC	0	0.0537	0.0185	0.0926	0.2439	0
Lactobacillus reuteri	0.068	0	0.0168	0	0	0
Pseudopropionibacterium propionicum	0	0	0.0142	0	0.0011	0
Sutterella wadsworthensis	0	0	0.0142	0	0	0
Citrobacter freundii	0	0	0.0139	0	0	0
Kocuria marina	0	0	0.0134	0	0	0
Bacteroides faecis	0	0	0.0112	0	0	0
Corynebacterium amycolatum	0	0	0.011	0	0	0
Bifidobacterium animalis	0	0	0.0102	0	0.0105	0
Citrobacter UC	0	0	0.0085	0	0	0
Lactobacillus johnsonii	0	0	0.0084	0	0	0
Porphyromonas gulae	0	0	0.0068	0	0	0
Staphylococcus haemolyticus	0	0	0.0059	0	0	0
Enterococcus avium	0	0	0.0056	0.0665	0.0232	0
Euzebya tangerina	0	0	0.0052	0.0004	0	0
Proteus mirabilis	0	0	0.0051	0	0.0179	0

Dermabacter porcinasus	0	0	0.0051	0	0	0
Eggerthellaceae UC	0.0914	0	0.005	0	0	0
Hyphomicrobium facile	0	0.0011	0.0035	0	0.0006	0
Bilophila wadsworthia	0	0	0.0034	0.0256	0.027	0
Paracoccus sphaerophysae	0	0	0.0034	0	0	0
Sphingomonas ginsenosidimutans	0	0	0.0034	0	0	0
Klebsiella UC	0	0	0.003	0	0	0
Enterococcus hirae	0	0.1054	0.0023	0.0353	0	0
Propionibacterium freudenreichii	0	0	0.0021	0.0064	0	0
TM7 sp. canine oral taxon 322	0	0	0.0015	0	0.0006	0
Blattella germanica	0.4265	0	0.0014	0	0	0
Olsenella UC	0	0	0.0014	0	0	0
Lactobacillus delbrueckii	0	0	0.0012	0	0	0
Aquisphaera giovannonii	0	0.0011	0.0011	0	0	0
Enterobacter roggenkampii	0	0	0.0011	0	0	0
Enterococcus casseliflavus	0	0	0.0011	0	0	0
Lactobacillus plantarum	0	0	0.0011	0	0	0
Parvimonas	0	0	0.0008	0	0	0
Corynebacterium mustelae	0	0	0.0007	0	0	0
Cutibacterium acnes	0	0.0031	0.0005	0	0	0
Pauljensenia hongkongensis	0	0	0.0003	0	0	0

Streptococcus gallolyticus	2.7429	1.66	0.0003	0	0	0
Bacteroides uniformis	0	0	0	0	0.5715	0
Bacteroides fragilis	0	0	0	0.1156	0.2124	0
Enterobacter kobei	0	0	0	0	0.1437	0
Sarcina ventriculi	0	0.0267	0	0	0.117	0
[Clostridium] bolteae	0	0	0	0	0.0857	0
Paeniclostridium sordellii	0	0.1221	0	0	0.0466	0
Klebsiella variicola	0	0.0333	0	0	0.0275	0
Enterobacter cloacae	0	0	0	0	0.0268	0
Others	--------	--------	--------	--------	--------	--------

Table S5. Pairwise comparison of Shannon Index between all time points in the FMT dogs and Healthy dogs.

**Pair**	***P*-value**	**FDR**
Healthy Dogs vs Screening	0.010	0.125
Healthy Dogs vs Endoscopy	0.019	0.125
Endoscopy vs Post-Treatment FMT	0.033	0.125
Screening vs Post-Treatment FMT	0.033	0.125
Inclusion vs Post-Treatment FMT	0.067	0.182
Screening vs Treatment FMT	0.073	0.182
Endoscopy vs Treatment FMT	0.128	0.275
Healthy Dogs vs Post-Treatment FMT	0.168	0.315
Healthy Dogs vs Inclusion	0.259	0.333
Endoscopy vs Inclusion	0.259	0.333

Inclusion vs Screening	0.259	0.333
Treatment FMT vs Post-Treatment FMT	0.267	0.333
Inclusion vs Treatment FMT	0.318	0.366
Endoscopy vs Screening	0.805	0.835
Healthy Dogs vs Treatment FMT	0.835	0.835

Table S6. Pairwise comparison of Shannon Index between all time points in the Placebo dogs and Healthy dogs.

**Pair**	***P*-value**	**FDR**
Healthy Dogs vs Endoscopy	0.021	0.269
Healthy Dogs vs Screening	0.036	0.269
Healthy Dogs vs Inclusion	0.071	0.354
Healthy Dogs vs Post-Treatment Placebo	0.131	0.492
Inclusion vs Screening	0.318	0.784
Treatment Placebo vs Screening	0.366	0.784
Treatment Placebo vs Endoscopy	0.366	0.784
Treatment Placebo vs Post-Treatment Placebo	0.548	0.997
Inclusion vs Endoscopy	0.710	0.997
Screening vs Endoscopy	0.710	0.997
Treatment Placebo vs Inclusion	0.731	0.997
Healthy Dogs vs Treatment Placebo	0.918	1.000
Inclusion vs Post-Treatment Placebo	1.000	1.000
Screening vs Post-Treatment Placebo	1.000	1.000
Post-Treatment Placebo vs Endoscopy	1.000	1.000

Table S7. Pairwise comparison of PERMANOVA analysis of Bray-Curtis dissimilarity index between all time points in the FMT dogs and Healthy dogs.

Pair	F-value	R-squared	P-value	FDR
Healthy Dogs vs Endoscopy	6.3326	0.15321	0.001	0.005
Healthy Dogs vs Inclusion	4.3151	0.10976	0.001	0.005
Healthy Dogs vs Screening	7.0097	0.16686	0.001	0.005
Healthy Dogs vs Treatment FMT	2.9234	0.077086	0.007	0.02625
Healthy Dogs vs Post-Treatment FMT	1.4006	0.043228	0.144	0.216
Endoscopy vs Inclusion	1.6934	0.12366	0.039	0.094286
Endoscopy vs Screening	0.53379	0.042588	0.826	0.924
Endoscopy vs Treatment FMT	1.6791	0.12275	0.044	0.094286
Endoscopy vs Post-Treatment FMT	1.457	0.15407	0.112	0.18667
Inclusion vs Screening	1.3185	0.098996	0.253	0.345
Inclusion vs Treatment FMT	0.4773	0.038254	0.873	0.924
Inclusion vs Post-Treatment FMT	1.0905	0.11996	0.349	0.43625
Screening vs Treatment FMT	1.7921	0.12994	0.041	0.094286
Screening vs Post-Treatment FMT	1.578	0.16475	0.107	0.18667
Treatment FMT vs Post-Treatment FMT	0.54473	0.06375	0.924	0.924

Table S8. Pairwise comparison of PERMANOVA analysis of Bray-Curtis dissimilarity index between all time points in the Placebo dogs and Healthy dogs.

Pair	F-value	R-squared	P-value	FDR
Healthy Dogs vs Treatment Placebo	3.425	0.092	0.004	0.015
Healthy Dogs vs Inclusion	4.825	0.121	0.001	0.005
Healthy Dogs vs Screening	6.902	0.165	0.001	0.005
Healthy Dogs vs Post-Treatment Placebo	1.809	0.055	0.050	0.094
Healthy Dogs vs Endoscopy	6.362	0.154	0.001	0.005
Treatment Placebo vs Inclusion	0.316	0.028	0.967	0.986
Treatment Placebo vs Screening	2.107	0.161	0.040	0.094
Treatment Placebo vs Post-Treatment Placebo	0.358	0.049	0.968	0.986
Treatment Placebo vs Endoscopy	2.072	0.158	0.008	0.024
Inclusion vs Screening	2.000	0.143	0.091	0.152
Inclusion vs Post-Treatment Placebo	0.481	0.057	0.938	0.986
Inclusion vs Endoscopy	1.949	0.140	0.045	0.094
Screening vs Post-Treatment Placebo	1.174	0.128	0.302	0.446
Screening vs Endoscopy	0.218	0.018	0.986	0.986
Post-Treatment Placebo vs Endoscopy	1.109	0.122	0.327	0.446

Table S9. Engraftment of Donor bacteria after FMT. ASV engraftment rates were calculated by dividing the number of ASVs shared between FMT recipients postFMT and their stool donors (excluding taxa shared between preFMT samples and donors) by the total number of ASVs in the donor sample (excluding any taxa shared with preFMT samples). 100% Engraftment would indicate that all of the donor ASVs with the capacity to engraft did indeed engraft.

**Dog ID**	**Number of ASVs that engrafted**	**Number of ASVs available to engraft**	**Engraftment Rate (%)**
S02	5	69	7.3
S03	25	68	36.8
S08	22	63	35
S17	14	56	25
S22	30	63	47.6
S24	29	66	44
S27	12	69	17.4

Table S10. Severity score of total histopathologic changes in the intestinal segments of TRE dogs in FMT and placebo groups.

Intestinal part	Group	Total histopathologic changes Severity score Median (range)
Duodenum	FMT (n= 5)	6 (3–7)
	Placebo (n= 6)	4 (2–15)
Ileum	FMT (n= 3)	8 (2–9)
	Placebo (n= 5)	6 (2–12)
Colon	FMT (n= 5)	5 (1–8)
	Placebo (n= 6)	5 (0–9)

Table S11. The diet information of each dog with TRE participated in the FMT/placebo trial.

Dog number	Group	Diet	
		Commercial food	Homemade food
S02	FMT	Specific CDW Food Allergy Management	-
S03		-	Rice, egg, chicken, salmon
S08		Royal Canin Gastrointestinal	-
S17		Golden Eagle Hypo-Allergenic	Fried beef and vegetables
S22		Oscar Chicken Meal	Chicken and minced meat
S24		Purina Pro Plan Veterinary Diets - EN Gastrointestinal	Cooked turkey mince, dark rice, pork heart, cooked cauliflower, oatmeal
S27		Brit Mono Protein	Turkey cutlet
S01	Placebo	Royal Canin Anallergenic	-
S04		Hill's Prescription Diet i/d	-
S05		Hill's Prescription Diet z/d	-
S07		-	Gluten-free oatmeal
S15		Royal Canin Gastrointestinal	-
S23		Hill's Prescription Diet Gastrointestinal Biome	Rice and chicken
S25		Purina Pro Plan Veterinary Diets - EN Gastrointestinal	-

Table S12. The individual data of the two FDM% measurements, their mean values, standard deviations, and coefficient of variation (CV%). FDM%: fecal dry matter percentage; SD: standard deviation

Dog number	Visit	Treatment group	FDM% measurement 1	FDM% measurement 2	Mean FDM%	SD	CV%
S02	Screening	FMT	38	36.1	37.05	0.95	2.56
S02	Inclusion		29	28	28.5	0.5	1.75
S02	Endoscopy		40	40.5	40.25	0.25	0.62
S02	Treatment		27.3	28.6	27.95	0.65	2.33
S03	Screening		49.1	50	49.55	0.45	0.91
S03	Inclusion		33.1	31.6	32.35	0.75	2.32
S03	Endoscopy		38.5	36	37.25	1.25	3.36
S03	Treatment		34.7	39.2	36.95	2.25	6.09
S08	Screening		38.9	34.6	36.75	2.15	5.85
S08	Inclusion		29.8	28.4	29.1	0.7	2.41
S08	Endoscopy		33.3	30.9	32.1	1.2	3.74
S08	Treatment		36.6	40	38.3	1.7	4.44
S08	Post-treatment		37.7	41.8	39.75	2.05	5.16
S17	Screening		32.1	29.2	30.65	1.45	4.73
S17	Inclusion		33.4	27.5	30.45	2.95	9.69
S17	Endoscopy		37	33.9	35.45	1.55	4.37
S17	Treatment		41.5	41.5	41.5	0	0.00
S22	Screening		33.9	33.3	33.6	0.3	0.89
S22	Inclusion		29.6	32.5	31.05	1.45	4.67
S22	Endoscopy		50	48	49	1	2.04
S22	Treatment		27.5	30.5	29	1.5	5.17
S22	Post-treatment		37.5	34.9	36.2	1.3	3.59
S24	Screening		27.6	29	28.3	0.7	2.47
S24	Inclusion		23.1	22	22.55	0.55	2.44
S24	Endoscopy		40.1	35.6	37.85	2.25	5.94
S24	Treatment		38	37.5	37.75	0.25	0.66
S27	Screening		41.4	38.5	39.95	1.45	3.63
S27	Inclusion		34.1	35	34.55	0.45	1.30
S27	Endoscopy		40.7	38.1	39.4	1.3	3.30
S27	Treatment		36	37.3	36.65	0.65	1.77
S27	Post-treatment		39	37	38	1	2.63

S1	Screening		37.8	38.1	37.95	0.15	0.40
S1	Inclusion		26.5	27	26.75	0.25	0.93
S1	Endoscopy		41.2	40.5	40.85	0.35	0.86
S1	Treatment		36.5	36	36.25	0.25	0.69
S1	Post-treatment		36	36.5	36.25	0.25	0.69
S4	Screening		42	48	45	3	6.67
S4	Inclusion		34.3	38	36.15	1.85	5.12
S4	Endoscopy		32.7	34.6	33.65	0.95	2.82
S4	Treatment		29.4	28.1	28.75	0.65	2.26
S5	Screening		42.3	41.1	41.7	0.6	1.44
S5	Inclusion		39	36	37.5	1.5	4.00
S5	Endoscopy		43.2	39.2	41.2	2	4.85
S5	Treatment		30.2	28.3	29.25	0.95	3.25
S5	Post-treatment	Placebo	43	42.9	42.95	0.05	0.12
S15	Screening		29.6	28.1	28.85	0.75	2.60
S15	Inclusion		22.4	21.3	21.85	0.55	2.52
S15	Endoscopy		42.1	38	40.05	2.05	5.12
S15	Treatment		34.1	36	35.05	0.95	2.71
S15	Post-treatment		40.4	41.5	40.95	0.55	1.34
S23	Screening		32.8	37.7	35.25	2.45	6.95
S23	Inclusion		27.3	26.5	26.9	0.4	1.49
S23	Endoscopy		37.7	36.5	37.1	0.6	1.62
S23	Treatment		27.5	27.8	27.65	0.15	0.54
S25	Screening		32.5	34.6	33.55	1.05	3.13
S25	Inclusion		31	29.6	30.3	0.7	2.31
S25	Endoscopy		36	37.3	36.65	0.65	1.77
S25	Treatment		25	26.1	25.55	0.55	2.15
